# Parkinson’s disease patient-specific neuronal networks carrying the LRRK2 G2019S mutation unveil early functional alterations that predate neurodegeneration

**DOI:** 10.1038/s41531-021-00198-3

**Published:** 2021-07-02

**Authors:** G. Carola, D. Malagarriga, C. Calatayud, M. Pons-Espinal, L. Blasco-Agell, Y. Richaud-Patin, I. Fernandez-Carasa, V. Baruffi, S. Beltramone, E. Molina, P. Dell’Era, J. J. Toledo-Aral, E. Tolosa, A. R. Muotri, J. Garcia Ojalvo, J. Soriano, A. Raya, A. Consiglio

**Affiliations:** 1 Department of Pathology and Experimental Therapeutics, Bellvitge University Hospital-IDIBELL, Barcelona, Spain; 2 Institute of Biomedicine (IBUB) of the University of Barcelona (UB), Barcelona, Spain; 3 Regenerative Medicine Program, Bellvitge Biomedical Research Institute (IDIBELL), and Program for Clinical Translation of Regenerative Medicine in Catalonia (P-CMRC), Hospital Duran i Reynals, Barcelona, Spain; 4 Cellular Fate Reprogramming Unit, Department of Molecular and Translational Medicine, University of Brescia, Brescia BS, Italy; 5 Sanford Consortium for Regenerative Medicine, UC San Diego, La Jolla, CA USA; 6 Instituto de Biomedicina de Sevilla (IBiS), Hospital Universitario Virgen del Rocío/CSIC/Universidad de Sevilla, Sevilla, Spain; 7 Department of Neurology, Hospital Clínic de Barcelona, Institut d’Investigacions Biomèdiques August Pi i Sunyer (IDIBAPS), University of Barcelona (UB), Barcelona, Spain; 8 Departments of Pediatrics and Cellular & Molecular Medicine, University of California San Diego, La Jolla, CA USA; 9 Department of Experimental and Health Sciences, Universitat Pompeu Fabra, Parc de Recerca Biomèdica de Barcelona, Barcelona, Spain; 10 Departament de Física de la Matèria Condensada, Universitat de Barcelona, Barcelona, Spain; 11 Universitat de Barcelona Institute of Complex Systems (UBICS), Barcelona, Spain; 12 Centre for Networked Biomedical Research on Bioengineering, Biomaterials and Nanomedicine (CIBER-BBN), Madrid, Spain; 13 Institució Catalana de Recerca i Estudis Avançats (ICREA), Barcelona, Spain

**Keywords:** Neuroscience, Diseases of the nervous system

## Abstract

A deeper understanding of early disease mechanisms occurring in Parkinson’s disease (PD) is needed to reveal restorative targets. Here we report that human induced pluripotent stem cell (iPSC)-derived dopaminergic neurons (DAn) obtained from healthy individuals or patients harboring LRRK2 PD-causing mutation can create highly complex networks with evident signs of functional maturation over time. Compared to control neuronal networks, LRRK2 PD patients’ networks displayed an elevated bursting behavior, in the absence of neurodegeneration. By combining functional calcium imaging, biophysical modeling, and DAn-lineage tracing, we found a decrease in DAn neurite density that triggered overall functional alterations in PD neuronal networks. Our data implicate early dysfunction as a prime focus that may contribute to the initiation of downstream degenerative pathways preceding DAn loss in PD, highlighting a potential window of opportunity for pre-symptomatic assessment of chronic degenerative diseases.

## Introduction

Parkinson’s disease (PD) is the most common neurodegenerative movement disorder, with an estimated prevalence in industrialized countries of 0.3% in the general population, which increases to 1.0% in people older than 60 years and to 3.0% in people older than 80 years^1^. Clinically, PD is characterized by classical motor syndrome linked to a progressive loss of dopamine-containing neurons (DAn) in the substantia nigra pars compacta, and disabling non-motor symptoms related to extranigral lesions. Current therapies for PD are symptomatic and do not limit the progression of disability with time. It has been proposed that early intervention might slow down or even stop disease progression, by preserving neurons from the undergoing irreversible neurodegeneration^1,2^. However, early treatment relies on early diagnosis, which unfortunately is especially complicated in the case of PD. Current diagnostic modalities in PD are based on the presence of motor symptoms, a stage at which up to 70% of DAn have been lost^3^. Even though pre-motor symptoms are known to precede clinical diagnosis of PD by as much as a decade, they are rather unspecific and unsuitable as stand-alone biomarkers of the disease^4^. Therefore, the identification of early diagnostic or progression markers of PD represents an urgent medical need.

Although the majority of PD cases are of unknown cause, so-called idiopathic PD, around 5% have been shown to have a genetic basis, with mutations in the *LRRK2* gene accounting for the largest number of patients of familial PD^5^. Interestingly, *LRRK2* polymorphisms are also considered a relevant genetic determinant for sporadic PD^6^, and LRRK2 function appears dysregulated in sporadic cases of PD^7^, even in the absence of *LRRK2* mutations/polymorphisms. These findings, together with the fact that PD associated with mutations in *LRRK2* (L2-PD) is clinically indistinguishable from sporadic PD, position LRRK2 as an essential player for understanding both genetic and idiopathic PD^8^. LRRK2 is a highly complex protein with multiple enzymatic domains, involved in a variety of intracellular signaling pathways and cellular processes such as cytoskeleton dynamics, vesicle trafficking and endocytosis, autophagy, reactive oxygen species, mitochondrial metabolism, and function of immune cells. However, the exact physiological role of LRRK2 and its implication for PD pathogenesis remains unknown^8^.

Of especial relevance for the investigation of early disease markers, transgenic mouse models of L2-PD display, before any events of neurodegeneration, an abnormally elevated excitatory activity and altered spine morphology in dorsal striatal spiny projection neurons^9^. Moreover, experimental models for other neurodegenerative conditions such as Alzheimer’s disease^10,11^ and amyotrophic lateral sclerosis^12^, have been shown to exhibit neuron hyperexcitability before the disease onset. It has also been demonstrated that the combination of PD with dementia often correlates with a disruption of both functional and effective connectivity in the cortex^13^. In contrast, the association of PD with depression correlates with disrupted functional connectivity between the median cingulate cortex and the prefrontal cortex and cerebellum^14,15^.

The development of induced pluripotent stem cell (iPSC) technologies enables the generation of patient-specific, disease-relevant, cell-based experimental models of human diseases. Importantly, iPSC-based models can recapitulate some of the earliest signs of disease, even at pre-symptomatic stages^12,16^. In this study, we used an experimental platform based on DAn-enriched neuronal cultures derived from L2-PD patients, their gene-edited isogenic counterparts, or from healthy individuals. Such cultures formed active neuronal networks, the functionality of which was analyzed by calcium imaging. After multiple iterations of experimental characterization and biophysical modeling of neuronal network behavior, we could identify early alterations in PD neuronal function that were not present in control networks, and that predated the onset of neuron degeneration.

## Results

### Generation and characterization of iPSC-derived DA neurons

A total of seven iPSC lines representing L2-PD patients and healthy aged-matched controls, along with gene-edited counterparts and fluorescent TH reporters, were used for the current studies (see Table 1 and “Methods” for further details). Some of these iPSC lines have been previously generated and fully characterized in our laboratories^17–19^, whereas two additional TH reporter lines were generated for this study (Supplementary Fig. 1).

**Table 1:** Summary of the healthy controls and patients used in this study.

Cell line code	Status	Code used in this study	Sex	Age at donation	Age at onset	LRRK2 mutation	Isogenic control
SP-11	Control	CTR	F	48		No	
SP-11TH	Control	CTR TH	F	48		No	
SP-12wt/wt	Isogenic Control	isoPD1	F	63		G2019S	LRRK2^G2019S^ corrected
SP-12	L2-PD	PD1	F	63	49	G2019S	
SP-12TH	L2-PD	PD1 TH	F	63	49	G2019S	
SP-12THwt/wt	Isogenic Control	Iso PD1TH	F	63		G2019S	LRRK2^G2019S^ corrected
SP-13	L2-PD	PD2	F	68	57	G2019S	

**Fig. 1:**
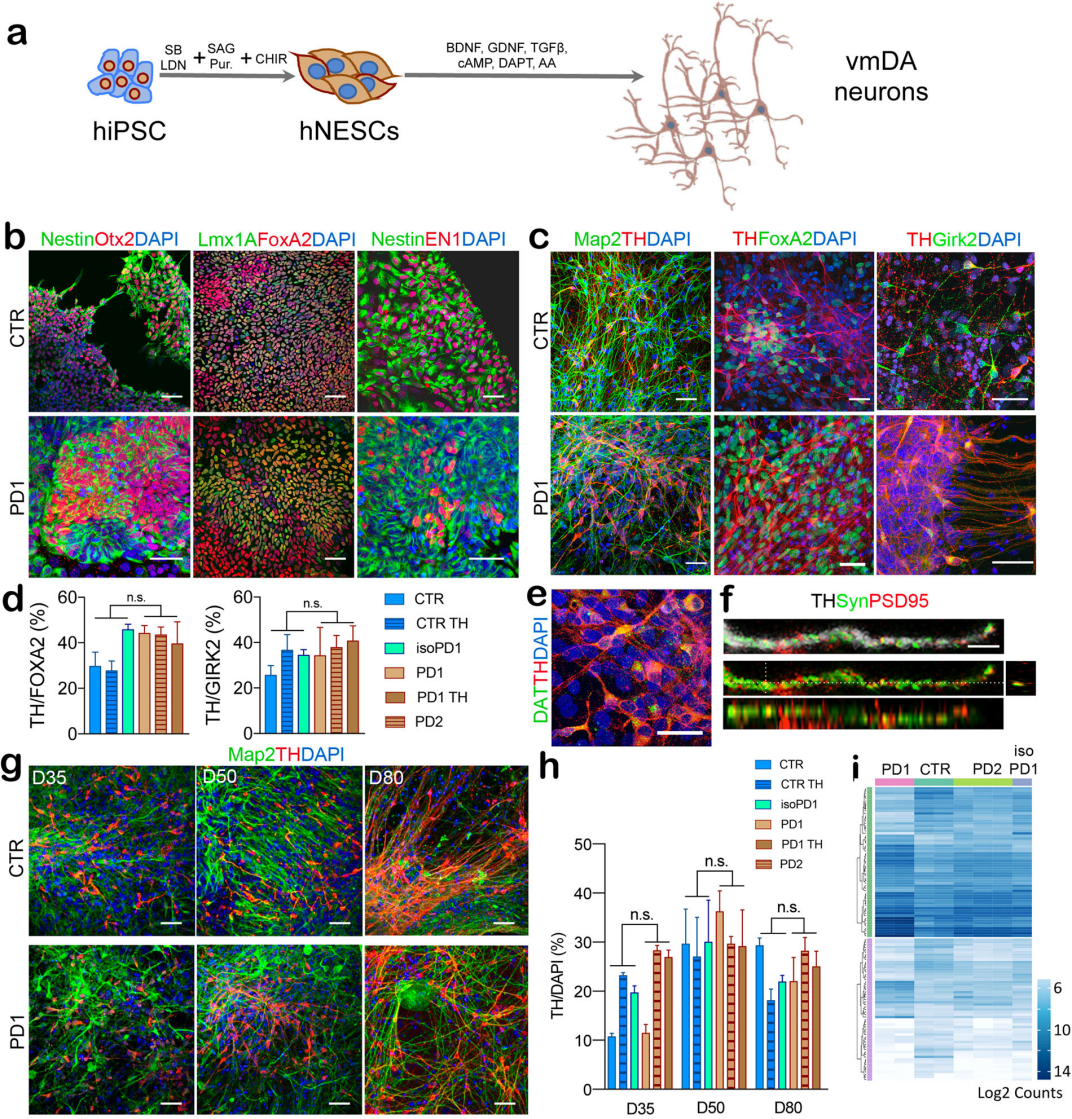
Generation of ventral midbrain (VM) dopaminergic neurons from human iPSCs. **a** Scheme showing VM dopaminergic neurons differentiation protocol. **b** Representative immunofluorescence (IF) images of CTR (SP11) and PD1 (SP12) NPCs at day 12 of the differentiation process. iPSC-derived neural cultures express floor plate progenitor markers, such as Lmx1A, FoxA2, Otx2, Nestin, and EN1. **c** Representative IF images of CTR (SP11) and PD1 (SP12) differentiated neuronal cultures expressing neuronal markers (MAP2, TH) and midbrain-type DA markers (FoxA2 and Girk2) at day 50. Scale bar is 50 μm. **d** Percentage of TH/FoxA2 and TH/Girk2 vmDAn at day 50 of all the lines [CTR (SP11), CTR TH (SP11 TH), gene-edited isoPD1 (SP12 wt/wt), PD1 (SP12), PD1 TH (SP12 TH), PD2 (SP13)]. A number of independent experiments *n* = 3. **e**–**f** Representative images of neuronal cultures at D50 expressing specific markers of maturation: **e** DAT (dopamine transporter) (scale bar, 50 μm); and **f** PSD95 (post-synaptic marker, red) and synapsin (synaptic marker, green). Orthogonal views show colocalization (scale bar, 10 μm). **g** Representative images of CTR (SP11) and PD1 (SP12) neuronal culture at D35-D50 and D80 showing the expression of TH and MAP2 markers at the different timepoints. **h** Percentage of TH+ cells over DAPI at the three different timepoints (D35-D50 and D80). **i** Heatmap of gene expression profiles of neuronal cultures of CTR (SP11), PD1 (SP12), PD2 (SP13), and gene-edited isogenic PD1 line (isoPD1) at D50, with dendrograms showing the strong similarities between independent experiments and the absence of clustering between control (CTR and isoPD lines, light brown) and PD (PD1 and PD2 lines, blue) conditions. Shown are transcripts with ≥2-fold change in expression, grouped as upregulated (50 transcripts, green bar) or downregulated (107 transcripts, purple bar). No statistically significant differences were found at p-Adj ≤ 0.1 when comparing control and PD conditions. Each type of culture was analyzed a minimum of 2 independent times, except for the gene-edited isogenic PD1 line, for which only one RNA preparation passed the quality control and could be analyzed.

iPSC differentiation toward DAn fate was performed using a modified version of the previously established midbrain floor-plate protocol^20^, which enabled the maintenance of differentiated cells for up to 10 weeks^21^. Briefly, we first cultured iPSCs on Matrigel with mTeSR medium until they reached 80% confluence, then we induced specification toward the ventral midbrain (VM) fate using a combination of knockout serum medium and neural induction medium (Fig. 1a). At day 12 post-plating (D12) the cells exhibited a homogeneous morphology and marker profile of VM floor-plate progenitors, expressing FOXA2+/LMX1A+ and the VM NPC markers such as OTX2 and EN1 paired with the neuroectodermal stem cell marker Nestin (Fig. 1b). These progenitors were then cultured in neuronal differentiation medium supplemented with growth factors including BDNF, GNDF, TGF-β, and DAPT, with the aim to foster neuronal differentiation and survival (Fig. 1a). At D50, the majority of the cells were positive for the dendritic marker MAP2, and quantitative immunolabeling for tyrosine hydroxylase (TH) and FOXA2 revealed that 30–40% of them were also committed to DA neuronal fate (Fig. 1c, d).

By the same day of differentiation (D50), about 35% of TH+ neurons also expressed the A9 domain-specific marker G protein-activated inward rectifier potassium channel 2 (GIRK2) (Fig. 1c, d), and displayed Dopamine Transporter (DAT), the essential marker of mature DAn (Fig. 1e). TH+ neurons also showed expression of pre-synaptic marker Synapsin and post-synaptic marker PSD95, indicating their capability to form synapses (Fig. 1f). Under these conditions, controls (CTR) and PD-iPSCs gave rise to VM DAn that were morphologically homogeneous and showed the expected features of mature VM DAn, including complex dendritic arborization (Fig. 1g). Although the protocol used was comparably effective in all iPSC lines analyzed, we found some variability in the number of VM DAn across lines at D35, ranging from 10 to 20% VM DAn ratio concerning all differentiated cells. This ratio did not depend on the presence or type of disease (Fig. 1h). Instead, it seemed related to the specific iPSC clone used and to the evolution at the early stages of differentiation.

After establishing equivalent DAn-enriched cultures from both control and patient iPSC lines, we evaluated whether there were any differences in cell viability between DAn derived from controls and patient iPSC lines. We first noted that control and L2-PD iPSC-derived DAn were indistinguishable, based on immunofluorescence (IF) images (Fig. 1g) up to 80 days in culture, the latest timepoint investigated. By counting the number of TH-expressing cells (Fig. 1h) we found no decline when cultured over time up to D80, strongly suggesting that DAn are not degenerating under these conditions (Fig. 1g, h). We also found no differences in the percentage of cells with pyknotic nuclei in patient lines compared to control lines (data not shown; D50: 12–15% in all the lines; D80: 15–20% in all the lines).

We next analyzed whether changes in transcriptome profiles suggestive of neurodegeneration appeared under these conditions. For this purpose, we measured the expression of a panel of 770 genes relevant for this process (NanoString Human Neuropathology Panel) in neuronal cultures differentiated from control and L2-PD iPSC at D50. Independent differentiation experiments from the same iPSC lines displayed highly similar gene expression profiles (Fig. 1i, Supplementary Fig. 2a), highlighting the robustness of the differentiation protocol. A comparison of gene expression levels between control and L2-PD cultures identified 157 differentially-expressed transcripts with ≥2-fold change (50 upregulated and 107 downregulated), but no differences reached statistical significance with p-Adj ≤ 0.1 (Supplementary Dataset), nor did they correlate with PD-related hallmarks (Supplementary Fig. 2b–d). Thus, even though previous studies have found overt signs of neurodegeneration upon long-term culture of DAn differentiated from L2-PD iPSC^17,18,22,23^, under the culture conditions used in the present work (that include neurotrophic factors), they appeared to form overall healthy neuronal cultures similar to the ones generated by control iPSC.

### Characterization of neuronal activity

**Fig. 2:**
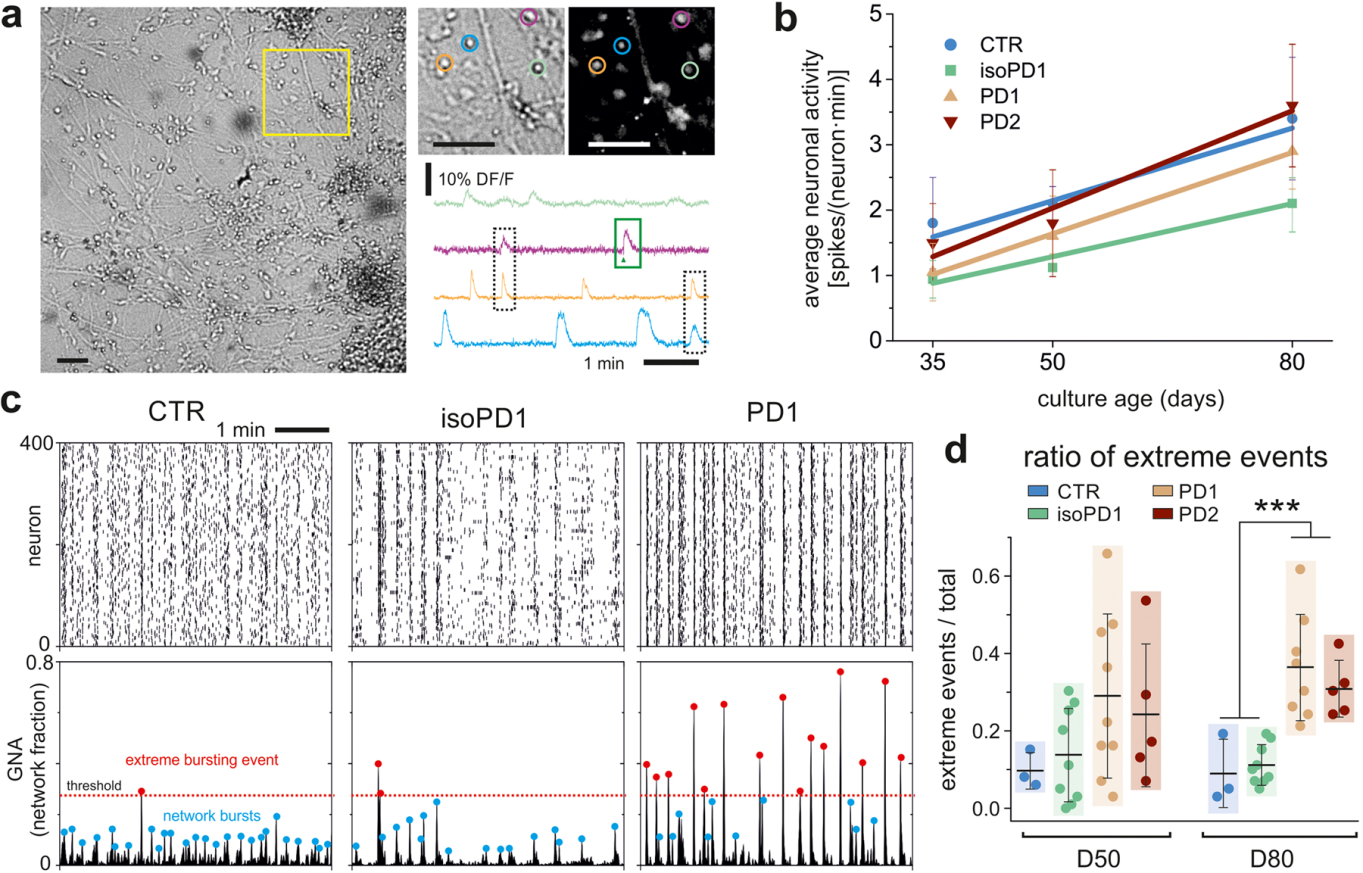
Network dynamics of vmDA neurons. **a** Representative image of a bright field recording at D80 (isoPD1) using the calcium imaging assay. Scale bar is 100 μm. Regions of interest were manually selected for each neuron (diameter 10 μm; color circles) to obtain the normalized calcium fluorescence time series of spontaneous activity, DFF (%) $\equiv 100 \cdot(F - F_0)/F_0$, with *F*_0_ the fluorescence signal of the neuron at rest. The green box illustrates the fast rise of fluorescence upon activation that procures the spiking onset time (arrowhead). The dashed black boxes illustrate coordinated neuronal activity that shape network bursts when several neurons are involved. **b** Average neuronal activity along maturation for the different cell lines. Data points are expressed as mean±SD. Trend lines are linear regressions. The number of cultures used in each condition and timepoint were: D35 (CTR: *n* = 3; isoPD1: 3; PD1: 4; PD2: 4); D50 (3; 9; 9; 5); D80 (3; 9; 8; 5). **c** Representative raster plots (top) and global network activity (GNA, bottom) for CTR (SP11), isoPD1, and PD1 (SP12) neuronal cultures at D80. Each plot shows 5 min of recording. Peaks in the GNA reveal network bursts (blue dots). Extreme bursting events (red dots) are those that are above a threshold (red dotted line) set as 95% confidence interval of CTR bursts’ distribution. CTR and isoPD1 networks show a relative low percentage of extreme events, which contrasts with the rich abundance of them in PD1 networks. **d** Ratio of extreme events for all studied cell lines at D50 and D80. The colored boxes are a guide to visualize the distributions, which show the mean ± SD and the individual realizations (dots). For panels (**b**) and (**d**), the number of independent experiments for each condition and timepoint are D35 (CTR: *n* = 3, isoPD1: 3, PD1: 4, PD2: 4); D50 (3, 9, 9, 5); D80 (3, 9, 8, 5). ***p < 0.001 (ANOVA with multiple comparison analysis).

Calcium activity recordings were then performed across all the seven independent lines to examine the functional maturity of the iPSC-derived neuronal networks after 35, 50, and 80 days of differentiation (D35-D50-D80) (Fig. 2a). We noted that our calcium fluorescence assay enabled tracking the behavior of ~500 neurons in the field of view with a high spatial and temporal resolution, allowing us to resolve single cells and their dynamic interactions.

Sharp increases in the fluorescence traces (Fig. 2a) revealed spontaneous neuronal activations, which were analyzed to extract the onset times of elicited action potentials. With these data we first computed the average neuronal activity along culture time (Fig. 2b) and observed that all lines evolved similarly, indicating that any anomalies in PD networks would depend on the structure of the activity patterns, rather than on the strength of activity itself. Thus, we next computed the Global Network Activity (GNA), defined as the fraction of neurons in the network that coactivated in a time window of 1 s. As shown in Fig. 2c, the GNA captures the level of neuronal synchronization present in the raster plot. For control cultures and also for the rescued isogenic PD line (left and central panels), collective activity encompassed synchronous activations of moderate size, between 10 and 40% of the network. In contrast, PD neuronal cultures (Fig. 2c, right panels) displayed a two-state dynamics, with strong whole-network synchronous events combined with quiescent intervals. Importantly, the distinct GNA patterns of control and PD neuronal cultures hint at the existence of intrinsically different network mechanisms in the two systems, which orchestrate a markedly different collective behavior. The GNA also informed that average neuronal activity by itself was not sufficiently informative to reveal alterations due to disease.

To quantify the differences between cell lines exposed by the GNA analysis, we next analyzed the amplitude of the GNA events and extracted those that exceeded a given threshold (Fig. 2c, red dotted line), termed ‘extreme event’. We observed that the ‘ratio of extreme events’, i.e., the number of large bursting episodes relative to all detected episodes, was much higher in PD lines than in CTR or in genetically-corrected isogenic control (isoPD) lines, particularly at late stages of maturation (D80) (Fig. 2d), which could render them more susceptible to stress by creating an overly rigid, synchrony-locked network despite their continued viability in culture.

### Functional connectivity of control and PD neuronal networks

**Fig. 3:**
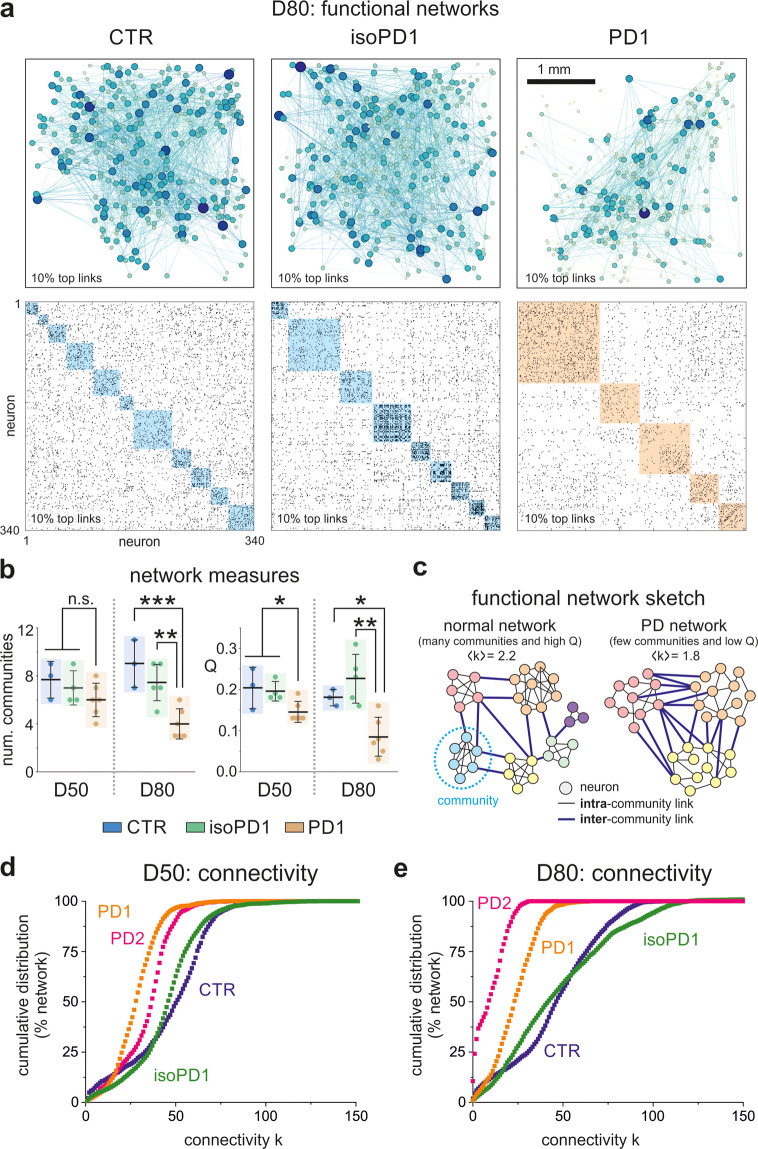
Functional connectivity of CTR, isoPD, and PD lines. **a** Top, Representative functional connectivity maps at D80 for CTR, isoPD1, and PD1 cultures. Dots are neurons and lines functional connections. The diameter of a dot and its opacity is proportional to the connectivity of the neuron it represents. Bottom, corresponding functional connectivity matrices. Black points are functional connections and colored boxes are functional communities. **b** Comparison of the number of communities and community statistic Q for the three lines at D50 and D80. PD1 networks are excessively integrated, with a relatively small number of communities strongly interconnected (low Q) as compared to CTR and isoPD1 networks. The colored boxes are a guide to visualize the distributions, which show the mean±SD and the individual realizations (dots). Number of independent experiments: D50 (CTR: *n* = 3, isoPD1: 4, PD1: 6); D80 (3, 5, 6). ****p < 0.001; **p < 0.01; *p < 0.05 (ANOVA with multiple comparison analysis). **c** Sketch of functional differences between normal and PD networks according to the data, with PD networks displaying on average a lower connectivity combined with fewer and excessively connected communities. **d** Cumulative distribution functions (CDF) of functional connections (in % of network) at D50 for CTR, PD1, PD2, and gene-edited isogenic PD1 line (isoPD1). The CDFs of the two PD lines exhibit a trend toward a lower connectivity as compared to CTR and isoPD1 ones, which are similar. **e** Corresponding distributions at D80. The two PD lines maintain their low connectivity profiles and strengthen their differences relative to CTR and isoPD1, which remain similar. The number of independent experiments used in panels (**d**) and (**e**) are D50 (CTR: *n* = 3, isoPD1: 4, PD1: 6, PD2: 5); D80 (3, 5, 6, 5).

The different structure of global activity of CTR, isoPD, and PD lines hints at the existence of distinct functional connectivity traits between them. To shed light on network functionality, we used transfer entropy (see “Methods” for details) to compute the functional connectivity among all pairs of active neurons in a given network. As shown in Fig. 3a (top panels), the functional networks displayed abundant connections, with a combination of short-range and long-range links that extended across the network. Even though the functional networks seemed similar in spatial organization, we observed significant differences between control and PD networks. The first difference was a lower density of connections in the PD line, suggesting an overall degradation of functionality. A second difference concerns the structure of functional communities, i.e., groups of neurons that tend to connect among themselves more strongly than with the rest of the network. As shown in the bottom panels of Fig. 3a, functional communities were abundant in CTR and isoPD cultures (highlighted blue boxes), indicating cross-talking of neurons in small groups, in agreement with the moderate size of collective activations. For PD cultures, however, the communities were much larger (orange boxes), indicating not only a failed formation of functional microcircuits, but a tendency toward excessively strong network synchronicity, as also seen in the raster plots.

These results were reproducible across realizations. As shown in Fig. 3b, PD networks exhibited a clear trend toward a small number of communities, which was accompanied by a tendency toward a stronger bond between these communities, i.e., an abnormal excessive integration. The latter was captured by the ‘community statistic Q’. The lower the Q value, the stronger the integration in the network. These differences appeared at D50 and substantially strengthened at D80. While CTR and isoPD cultures showed a moderate integration, PD cultures exhibited an abnormally strong integration. The combination of these results is summarized in Fig. 3c, which depicts two toy networks with the same number of neurons but with different functional organization. The normal, healthy network is characterized by a high average connectivity and well-defined small communities, while the PD network is characterized by a lower average connectivity and large, strongly linked communities that effectually almost shape a unique structure.

To complete the functional analysis, we compared in more detail the statistics of functional connections. Figure 3d, e shows, for D50 and D80 stages of development, the cumulative distribution function of connections, CDF(k), for control, isoPD, and PD lines. This distribution portrays the probability that a neuron in the network has a number of connections less or equal than k. For D50, all distributions were similar and relatively close to one another, illustrating that they originated from similar dynamics, namely a combination of individual activity and network bursting. The distributions also showed a subtle growth, indicating that weakly connected neurons were rare. However, the PD distributions at D50 revealed a tendency toward a lower connectivity. Indeed, although the distributions were similar in shape, an analysis of the distance between distributions (Kullback–Leibler divergence and Kolmogorov–Smirnov test, Supp. Fig. 3) showed statistical differences among them, indicating that D50 could be the characteristic timepoint at which functional alterations in PD lines start to be detectable. The differences among distributions accentuated at D80, with PD cultures exhibiting a more pronounced trend toward a lower connectivity (a sharp increase of CDF(k) at low k values) that markedly departed from CTR and isoPD cultures. Interestingly, there were no statistically significant differences between CTR and isoPD cultures at this timepoint, a result that suggests the successful rescue of affected cell lines through correction of the *LRRK2* mutation by gene edition.

In light of these results, we considered the control network as the reference for a healthy development and function, and the departure from it as a signature of the pathology. Thus, we hypothesize that the *LRRK2* mutation undermines the development of neuronal circuitry to such an extent that it alters collective network activity and functional organization.

### Contrasting dynamics of TH and non-TH neurons in control and PD lines

**Fig. 4:**
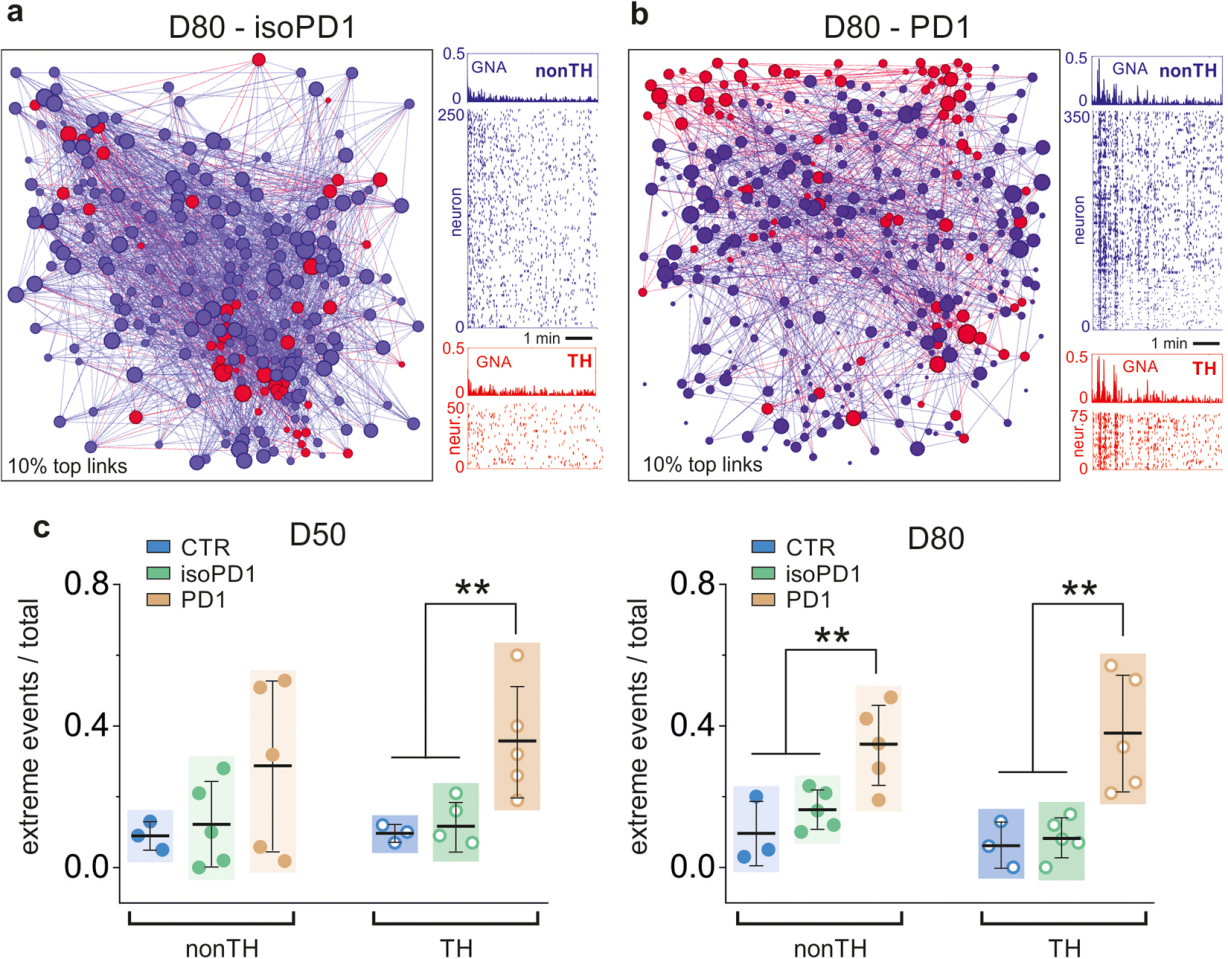
Functional connectivity and dynamics of TH+ and non-TH+ neurons. **a** Left, representative functional network of an isoPD1 culture at D80, signaling the location of TH+ neurons (red) and non-TH+ neurons (blue). The diameter of a dot is proportional to the connectivity of the neuron it represents. Only 10% of the connections are shown for clarity. Right, corresponding raster plots of the two populations. **b** Corresponding analysis for a PD1 culture, in which the non-TH+ subpopulation exhibits a much stronger synchronous behavior as compared to controls. **c** Ratio of extreme events for each subpopulation of neurons at two maturation stages, D50 and D80. PD cultures show in general a higher ratio of extreme events as compared to controls. The non-TH+ population in the PD network at D50 shows a strong variability in the ratio of extreme events across realizations, indicating the onset of malfunction. The same population at D80 is dominated by extreme events that reflect the strong synchronous behavior. The colored boxes are a guide to visualize the distributions, which show the mean ± SD and the individual realizations (dots). The number of independent experiments in panel (**c**) are D50 (CTR: *n* = 3, isoPD1: 5, PD1: 5); D80 (3, 5, 5). ***p < 0.001; **p < 0.01; *p < 0.05 (ANOVA with multiple comparison analysis).

To investigate the origin of the functional impairment found in PD DAn, we took advantage of the genetic TH-reporter tool created in our lab^19^. This reporter allows identifying TH and non-TH neuronal populations in the networks and analyzing their functional characteristics separately. Figure 4a, b exemplifies such a construction for representative isoPD and PD networks at D80, in which two neuronal layers, one corresponding to TH neurons (red) and another one corresponding to non-TH neurons (blue), interact functionally. The spontaneous activity of each subpopulation of neurons at D80 is shown in the accompanying raster plots. A comparison of the activity patterns between isoPD and PD cultures reveals the strong contrast in their dynamics. While the non-TH population in isoPD cultures shows a sustained activity with weak collective events, PD cultures show strong synchrony episodes that extend to both subpopulations.

In addition, as shown in Fig. 4c, a comparison of the ratio of extreme events for TH and non-TH subpopulations indicates that, on average, PD cultures exhibited a higher number of extreme events in both subpopulations when compared to controls. However, along development, TH and non-TH ratios for controls were similar at D50 and D80, whereas the ratios for the PD line switched from mostly TH at D50 to mostly non-TH at D80. Altogether, these results unfold an abnormal subpopulation dynamics in PD networks, with an overall excessive bursting and a reversing leadership of TH and non-TH subpopulations along development. We also noted that PD cultures at D50 showed important variability among realizations, with extreme events in the non-TH population being absent in some experiments and abundant in others. Given this variability, we hypothesize that the D50 timepoint might mark the onset of structural alterations that later translate into dynamic and functional deficits.

Since about 70% of the neurons in our networks are non-TH (Fig. 1g, h), the above results suggest that, in healthy control cultures, non-TH neurons drive spontaneous activity in the network. In contrast, TH neurons play a regulatory yet essential role by facilitating the coexistence of small neuronal coactivations and whole-network bursting events. This regulatory role appears to be lost in PD cultures, in which TH neurons frame spontaneous activity patterns with excessive synchrony that translate into an abundance of extreme bursting episodes. We, therefore, hypothesize that the *LRRK2* mutation alters the balance between neuronal subpopulations by degrading the physical coupling among neuronal types.

### An in silico model captures the dynamic alterations in PD networks

Next, we carried out numerical simulations to better understand the impact of TH+ cells structural failure on PD network dynamics. Specifically, we evaluated whether a reduction in TH cells connectivity was sufficient to switch global network dynamics from balanced to excessively synchronous.

**Fig. 5:**
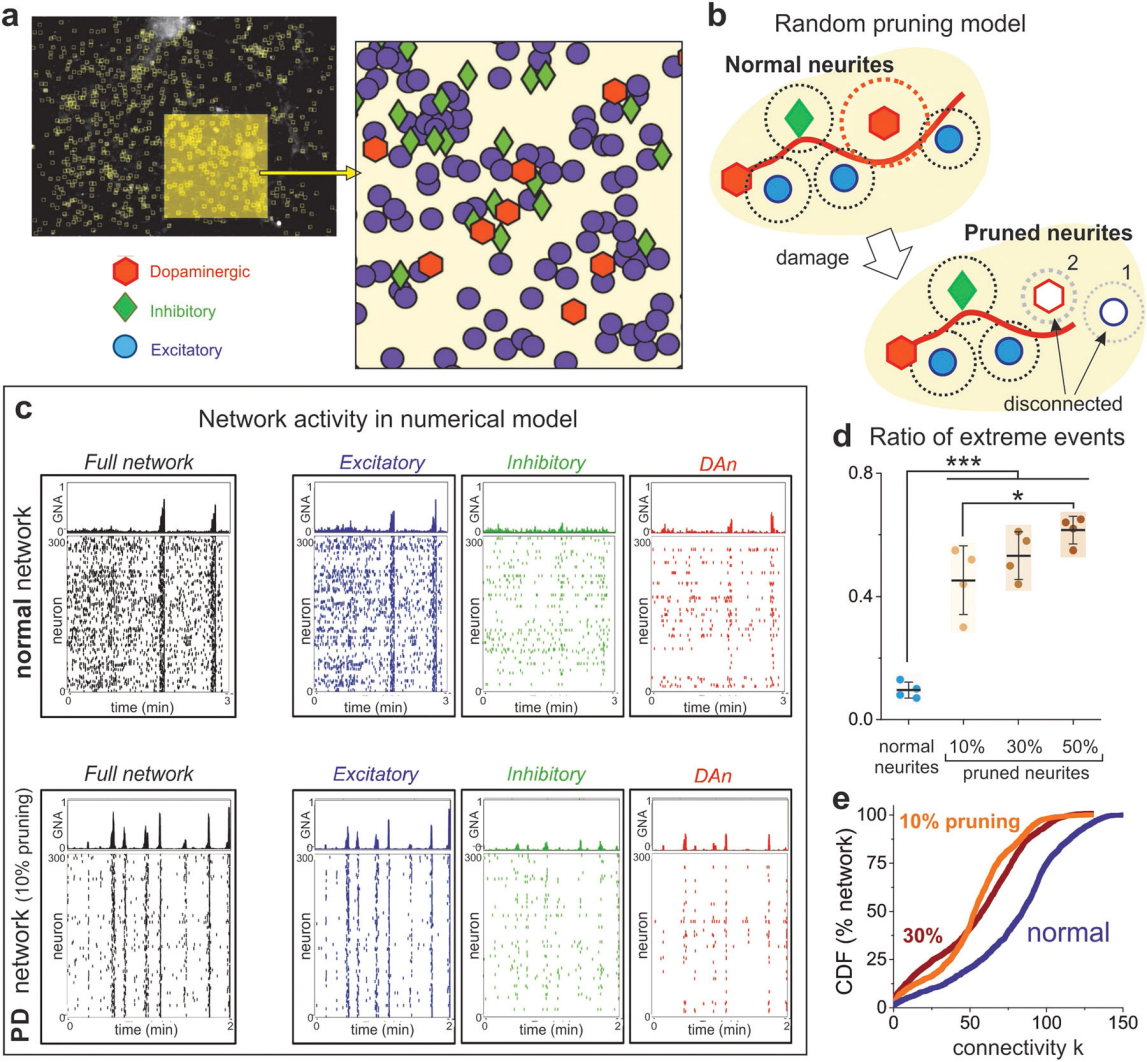
Numerical simulations of normal and PD networks. **a** Representative CTR culture at day 80, used as a reference for the spatial allocation of neurons in the numerical model. The enlarged area depicts a detail of the neuronal spatial arrangement. 300 neurons are used in the simulation, and are randomly assigned as dopaminergic (DA, red, 25% of network), inhibitory (green, 20%), and excitatory (blue, 55%). **b** Random pruning algorithm, in which PD damage is simulated by a shortening of either axons (red curve) or dendritic arbor (dotted red circle) in DA neurons (red hexagons). In the sketch, normal connectivity is established when the dendritic arbor of any neuron (dotted circles) intersects an axon. Neurite pruning disconnects neurons either because of axonal shortening (neuron #1) or dendritic loss (neuron #2) in DAn. **c** Raster plots of simulated normal and PD networks, with the latter corresponding to an axonal pruning on 10% of DA neurons, each one losing about 80% of connections. In the raster plots, the left panels show the dynamics of the entire network, whereas the right panels show the dynamics in each subpopulation. All raster plots range from 0 to 300, but the number of active neurons in each plot vary according to the population monitored. PD simulations show a markedly synchronous behavior of the excitatory population that translates onto the entire network. **d** Ratio of extreme events for different percentages of pruned DAn subpopulation, showing that the presence of extreme bursting events increases as network affectation progresses. The colored boxes are a guide to visualize the distributions, which show the mean ± SD and the individual realizations (dots). The number of individual simulations is *n* = 4 for each condition. ***p < 0.001; *p < 0.05 (ANOVA with multiple comparison analysis). **e** Cumulative distribution of functional connections (CDF) for two PD networks (at 10 and 30% pruning) and a normal, non-pruned network. The distributions show the same trend as in the experiments, with PD departing from normal toward a low degree functional connectivity scenario.

We first reproduced the behavior of control networks. As shown in Fig. 5a, we used the same neuronal spatial arrangement of the cultured networks and considered a mixed population of 55% excitatory non-TH neurons, 25% excitatory TH+ neurons, and 20% inhibitory neurons. These values were selected to concord with both the typical 80% excitation of cortical circuits in vitro^24^ and the TH/DAPI ratio found in our cultures (Fig. 1). Neuronal dendritic trees and axons were then incorporated according to realistic biological rules, so that a connection was established whenever an axon crossed the dendritic tree of a neuron (Fig. 5b, top). Once the network structure was set, dynamics was incorporated through an extended Hodgkin–Huxley model with parameters adjusted to replicate the activity in controls. As shown in the top panels of Fig. 5c, normal network dynamics was qualitatively similar to the experiments shown in Fig. 2c (left and center) and characterized by an intense background activity in combination with coordinated activity episodes. The inspection of the different populations (Fig. 5c, top, right panels) shows that non-TH neurons were also the drivers of network dynamics. It should be noted that inhibition was necessary in the simulations to ensure a sufficiently high spontaneous activity.

To explore PD dysfunction, we used the same control networks and modeled the shortening of neurites in TH neurons, effectually reducing the connectivity probability in the TH population (Fig. 5b, bottom). Such a construction signified that a single TH cell lost about 80% of its connections. We then explored the minimum fraction of TH cells that had to be affected to observe an impact on the network dynamic. As shown in the bottom panels of Fig. 5c, PD simulations were characterized by an exceedingly synchronous behavior that captured well the experimental observations of PD cultures (Fig. 2c, right), with a much lower background activity and a similar dynamic in both TH and non-TH populations. Simulations also demonstrated that the affectation of ~10% TH cells sufficed to drive the networks toward a chronic bursting behavior with an abundance of extreme, whole-network synchronous events (Fig. 5c). As shown in Fig. 5d, we compared three different ratios of affected TH neurons. An increase in this ratio mimics disease progression, i.e., developmental time in the experiments. The results show that the ratio and occurrence of extreme events are similar for different damage rates, possibly because the remaining excitatory and inhibitory neurons are sufficiently large to maintain activity. Interestingly, these results also indicate that a small damage suffices to drive the network toward an excessively synchronous scenario. Finally, an analysis of functional connectivity showed that the model also reproduces well the experimental observations, with a tendency for simulated PD cultures to shift toward lower connectivity states (Fig. 5e).

### TH+ neurons carrying *LRRK2* mutation feature a lower number of dendrites

**Fig. 6:**
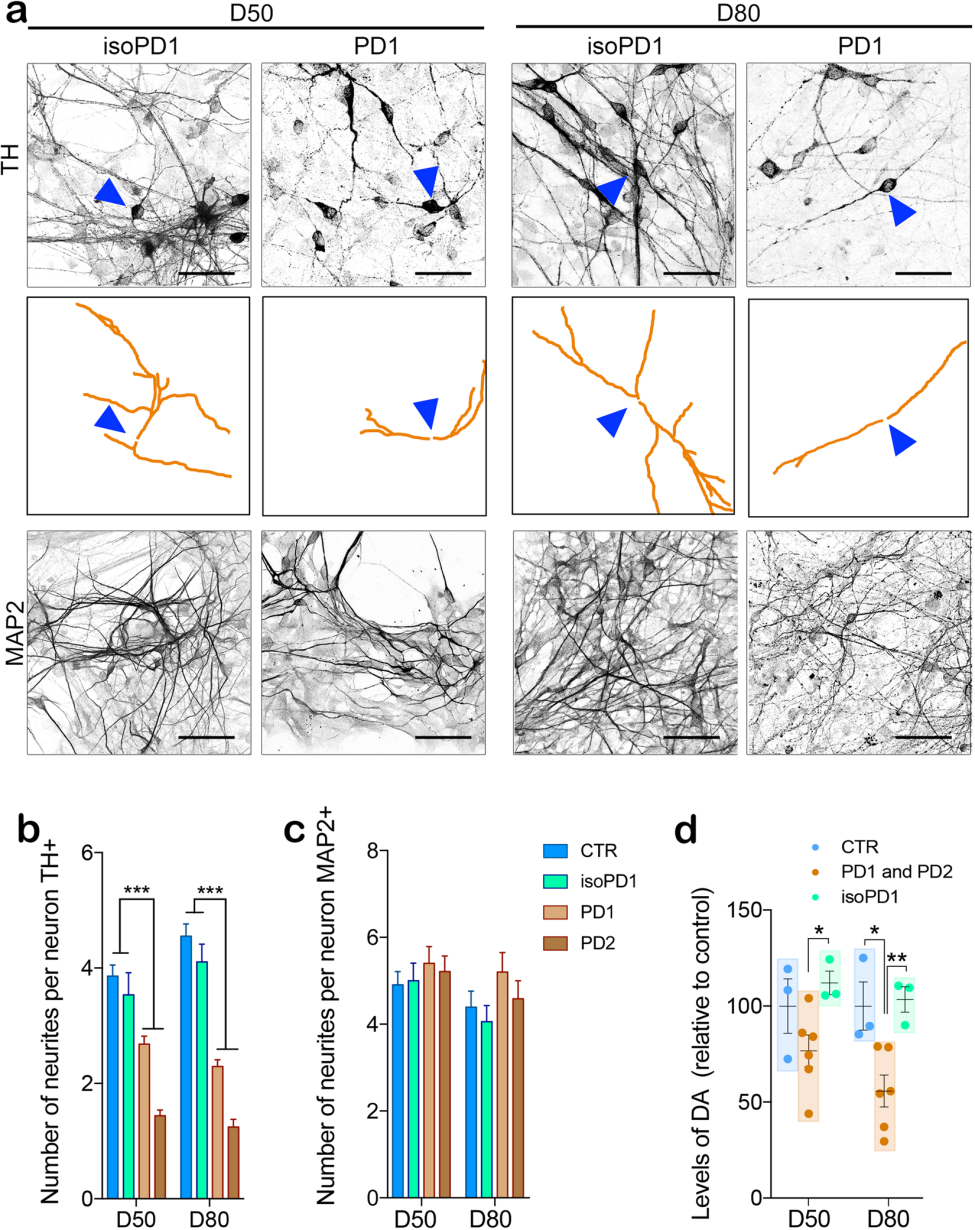
Biological confirmation of the in silico hypothesis. **a** Gene-edited isoPD1 (SP12 wt/wt) and PD1 (SP12) TH+ neuronal processes are traced (orange) to determine the number and the structure of neurites at D50 (left panels) and D80 (right panels). The top panels for each cell line show the original image of TH+ neurons. A blue triangle highlights a representative traced neuron shown in the middle panels. The bottom panels for each cell line show MAP2+ neuronal processes. Scale bar is 50 μm in all images. **b** The quantification of the number of TH neurites shows significant differences between CTR and PD1 both at D50 and D80. **c** No differences are detected on the number of MAP2 neurites. **d** Levels of released DA measured in culture media from CTR (SP11), PD1 (SP12), PD2 (SP13), and isoPD1 (SP12 wt/wt) at D50 and D80 of differentiation, relative to CTR levels. *p-value < 0.05; **p-value < 0.01; ***p-value < 0.001 (ANOVA with multiple comparison analysis). Number of independent experiments *n* = 3.

To verify the prediction of the in silico model above and expose physical alterations in the circuitry of PD lines, we investigated the morphology of TH neurons in CTR, isoPD, and PD neuronal cultures at D50 and D80 (Fig. 6a and Supplementary Fig. 4a). We found that DAn differentiated from PD iPSC lines showed a lower number of TH neurites compared to those derived from CTR or isoPD lines (1.2 ± 0.1 neurites for PD *vs*. 4.5 ± 0.2 for CTR and 4.1 ± 0.3 for isoPD, Fig. 6b). In addition, the number of neurites in CTR and isoPD cultures increased along with development while it decreased in PD cultures, indicating a progressive deterioration of network structure for the latter (Fig. 6b). In contrast, the number of neurites in MAP2+ neurons from control, isoPD, and PD lines did not show any significant differences (Fig. 6c), confirming that the dynamic and functional deficits of PD lines are localized to the TH subpopulation, and that these cells experience a gradual structural failure in the form of neurite loss.

Next, we evaluated DA release in the media collected from CTR, isoPD, and PD neuronal cultures. Supernatants of PD cultures revealed decreased dopamine levels at D50 and D80 compared with those of CTR and isoPD cultures (Fig. 6d), indicating that the reduction in TH+ neurites has functional consequences on DA release. Finally, to investigate whether the early sign of overall alteration in the network structure and dynamics could contribute to neurodegeneration, we maintained iPSC-derived DAn over culture times longer than D80. Interestingly, in aged (110 days, latest timepoint analyzed) cultures, PD DAn showed morphological alterations, including reduced number and length of neurites, and significantly decreased cell survival compared with isoPD DAn (Supplementary Fig. 5a–d). In contrast, neuronal degeneration was not evident in non-TH+ cells, as judged by the percentage of MAP2+/TH− neurons in the neuronal cultures (Supplementary Fig. 5e).

## Discussion

Since early diagnosis of PD is expected to dramatically improve the outcome of therapies under current development, in the present study we interrogated a human neuronal cell-based model of PD for the earliest detectable functional alterations. We found that DA neurons derived from iPSC representing healthy individuals or PD patients harboring *LRRK2* mutation developed appropriate physiological characteristics forming complex and mature networks during the differentiation process. However, PD neuronal networks developed abnormal hypersynchrony at the latest timepoint analyzed (D80), in contrast with healthy or gene-edited isogenic PD networks. These new data from PD-affected human DAn indicate that early dysfunction may contribute to the initiation of downstream degenerative pathways that ultimately lead to DAn loss in PD. A general limitation of human iPSC-based disease modeling strategies that should be taken into account when interpreting the results of our studies is the notorious variability described among iPSC lines and clones^25^. To rule out the impact of interline/interclone variability in our findings, we used several iPSC lines and iPSC clones to represent each experimental condition. Specifically, controls included two independent iPSC lines generated from two independent individuals, and a total of four iPSC clones, whereas the PD condition was similarly represented by two independent iPSC lines generated from two independent individuals, and a total of three iPSC clones (Table 1). Moreover, the confirmation of our findings in neuronal cultures differentiated from gene-edited isogenic controls of PD iPSC provides further reassurance that the alterations identified in PD samples in our studies are indeed related to the disease condition, rather than reflecting the specific iPSC line/clone utilized.

Our previous work showed that PD patient-specific iPSC-derived DAn are particularly susceptible to undergo neurodegeneration upon long-term culture^17,18,23,26^. In those studies, DAn were differentiated from iPSC and then maintained in culture in the absence of neurotrophic factors, which resulted in evident signs of DAn neurodegeneration only in PD samples, after 75 days in culture. For the present study, we used a different protocol for the generation and maintenance of iPSC-derived DAn in culture, which included neurotrophic factors and allowed comparing the function of mature DAn from PD and control iPSC in the absence of neurodegeneration for up to 80 days. Following these conditions, we used a calcium fluorescence imaging assay to monitor the functional neuronal activity of control and L2-PD iPSC-derived DAn at different timepoints of differentiation. We found that DAn derived from control iPSC exhibited an increase in both the number of spontaneous activity events and the number of bursting episodes, i.e., network-spanning synchronous activations. Such a trend is the one that could be expected from maturing healthy iPSC-derived neurons in vitro^27^. Conversely, L2-PD iPSC-derived DAn showed a two-state dynamic completely different from controls, characterized by strong synchronous events combined with quiescent intervals. The dynamics of PD cultures suggests that the structure of collective activation —but not the average individual neuronal activity—was the critical feature of PD malfunctioning neuronal behavior. This apparent sign of functional alterations was only displayed by the diseased neurons at a late timepoint. Early in development, both control and PD neuronal cultures showed similar functional behavior, indicating that there is a defined period of time in which PD network development starts to degrade and functional deficiencies emerge. The proportion of control iPSC-derived DAn in each of the cell lines remained unchanged along the ten weeks of culturing. Thus, despite remaining viable in culture, PD-affected DAn failed at procuring the necessary structural support for a rich collective dynamic and brought the network toward an excessively synchronous state.

We introduced the connectivity probability of the TH population into a biophysical model to address the role of neurite connectivity. Numerical simulations of the model revealed that neurite density loss in DAn was among the first causes of dynamic and functional alterations. A random neurite loss in 10% of DAn sufficed to render a bursting-dominated dynamic and an excessively connected functional network. Our experimental data confirmed the reduction of neurites specifically in DAn of PD neuronal cultures. Thus, even though the culture conditions used in the present studies prevented the appearance of overt signs of PD DAn neurodegeneration observed in previous works using more standard culture conditions^17,22,28^, they could not prevent a slight reduction in PD DAn neurites. Therefore, it is important to note that this phenotype appears to be independent from differentiation and culture methods, depends on the presence of the *LRRK2*^*G2019S*^ mutation (inasmuch as it is absent in gene-corrected isogenic PD DAn), and underlies the functional alterations in overall network dynamics. It is tempting to speculate that this observation might be related to the recent finding that the LRRK2-G2019S mutated protein interferes with microtubule-based motors^29^.

Previous studies of PD patients^15^ and animal models of the disease^9^ have reported early hyperexcitability of DAn and corticospinal neurons of the motor cortex. In our study, we demonstrate that DAn derived from patient iPSC harboring *LRRK2* mutations exhibit hyperexcitability in culture. This increased activity might contribute to trigger a cascade of excitotoxic disease mechanisms involving pathological changes in Ca^2+^ handling and the eventual activation of cell death pathways. In contrast, recent work supports a link between hyperexcitability and neuroprotection^30,31^, in particular when hyperexcitability is induced via reduction of neurite density and a consequent lack in connections and communication between neurons. This connectivity loss may trigger compensatory mechanisms in which the neurons create an aberrant structural and functional connectivity that can be pointed as an early marker of pathology^32,33^. To determine the actual role of hyperexcitability in PD, it will be necessary for future studies to examine the effects of finely controlled manipulations of excitability on human DAn.

Thus, dysfunction of DAn physiology appears to precede the functional alterations that are then spread in the overall culture, placing DAn dysfunction as an early sign of overall alteration and neurodegeneration. Moreover, we directly connected this alteration to a reduction in the neurite arborization using both experimental data and in silico analysis. Taken together, these findings highlight the importance of addressing early changes in mechanisms underlying spike generation at the DAn soma when considering disease pathogenesis and potential treatment strategies for PD. Furthermore, our findings show the usefulness of sensitive physiological studies of human iPSC-derived DAn for future work aiming to develop new diagnostic tools and therapeutics for PD.

## Methods

### iPSC lines and gene editing

The parental iPSC lines used in our studies were previously generated and fully characterized^17–19^. The generation and use of human iPSCs in this work were approved by the Spanish competent authorities (Commission on Guarantees concerning the Donation and Use of Human Tissues and Cells of the Carlos III National Institute of Health). All procedure adhered to internal and EU guidelines for research involving derivation of pluripotent cell line. All subjects gave informed consent for the study using forms approved by the ethical Committee on the Use of Human Subjects in Research at Hospital Clinic in Barcelona. The iPSC lines used in this study include one iPSC line obtained from a healthy donor (SP11) and two lines obtained from PD patients carrying the LRRK2 G2019S mutation (SP12 and SP13). From these original lines, isogenic controls solely differing in the presence of the LRRK2 G2019S mutation were obtained by correcting the LRRK2 mutation in the SP12 iPSC line. Expanded subject information, cell characterization, and technical details of the original iPSCs are described in Table 1.

### Generation of CRISPR/Cas9 plasmids and donor template for homology-directed repair

For correcting the *LRRK2* mutation, LRRK2 G2019S mutant SP12 iPSC were edited using TALENs. The CRISPR/Cas9 plasmid pSpCas9(BB)-2A-GFP (PX458) was a gift from Dr. Feng Zhang (Broad Institute, MIT; Addgene plasmid #12345)^34^. The original pCbh promoter was exchanged for the full-length pCAGGS promoter to achieve higher expression levels in hiPSCs. Custom guide RNAs were cloned into the BbsI sites as annealed oligonucleotides. The donor template for HDR was generated using standard molecular cloning procedures. Briefly, for *TH* donor template, homology arms were amplified from genomic DNA and verified by Sanger sequencing. Resulting sequences matched those of the reference genome GRCh38. The homology arms were inserted into the KpnI-ApaI (5′HA) and SpeI-XbaI (3′HA) sites of pBS-SK ( − ). The sequence coding for the P2A peptide was added to mOrange with the primers used to amplify the gene and the PCR product was inserted into the ApaI-XhoI sites of the pBS-5′HA-3′HA plasmid. Finally, LoxP-pRex1-Neo-SV40-LoxP was amplified from the aMHC-eGFP-Rex-Neo plasmid (gift from Dr. Mark Mercola; Addgene plasmid #21229)^35^ and inserted between the XhoI-SpeI sites of the plasmid. For *LRRK2* donor template, homology arms were amplified from genomic DNA of a wild-type donor and verified by Sanger sequencing. The homology arms were inserted in the KpnI-XhoI (5′HA) and SpeI-NotI (3′HA) sites of pBS-SK ( − ). LoxP-pRex1-Neo-SV40-LoxP was inserted in a second cloning step between the SalI-BamHI sites.

### Gene edition in iPSC

To generate the TH-mOrange hiPSC reporter cell line, cells were transfected with the HDR template and a Cas9- and gRNA-encoding plasmid; the latter overlapping the *TH* gene stop codon. In total, 800,000 iPSCs were seeded in 10-cm plates the day before transfection. iPSCs were co-transfected with 6 µg of CRISPR/Cas9 plasmid and 9 µg HDR template using FuGENE HD (Promega) at a 1:3 DNA to reagent ratio. Cells were plated in selection medium containing 50 µg/mL G418 (Melford Laboratories Ltd., Ipswich, UK) and maintained for 2 weeks until resistant colonies could be screened. At that time, one-half of each resistant colony was manually picked and site-specific integration was verified by PCR. To correct the LRRK2 G2019S mutation in the SP_12 iPSC line, cells were transfected with the wild-type HDR template and a Cas9- and gRNA-encoding plasmid whose gRNA overlapped the insertion site for the selection cassette. Transfection, clone selection, and subsequent screening were conducted as described above. To excise the selection cassette, edited iPSCs were transfected with a CRE recombinase-expressing plasmid, gifted from Dr. Michel Sadelain (Sloan Kettering Institute; Addgene plasmid #27546)^36^. At 48 h post-transfection, cells were dissociated and seeded at clonal density on a feeder layer of irradiated human fibroblasts. When colonies attained a certain size, they were picked and subcultured in independent Matrigel-coated wells. Cells were sampled and checked for cassette excision by PCR and Sanger sequencing. Those clones in which the cassette was excised were expanded, cryopreserved, and karyotyped. Expanded information regarding oligonucleotides used during gene editing procedures are listed in Supplementary Table 3 in Supplementary Information.

### iPSC differentiation into vm DA lineage

Directed differentiation of iPSC onto ventral dopaminergic neurons (DAn) was carried out following a previously published protocol^20^, with minor modifications. Briefly, iPSCs were cultured in mTeSR commercial medium until they reached 80% confluence. Ventral midbrain induction was then forced by switching to SRM medium (KO-DMEM,15% KO serum, 1% P/S, 1% glutamine, 1%NEAA, 0.1% bet- mercaptoethanol) with SB Tocris 1614 (a selective inhibitor of the growth factor TGF-b), LDN193189, Stemgent 04-0074 (BMP inhibitor) to inhibit the dual SMAD pathway, SAG and Purmorphamine, Calbiochem 540220 (SHH pathway activators) to induce neuroepithelial stem cells formation and proliferation. The medium was next changed to Neurobasal with 1% P/S, 1% N2, and 2% B27-VitA and CHIR99021, Stemgent 04-0004 (CHIR), a potent GSK3B inhibitor known to strongly activate WNT signaling that induces LMX1A in FOXA2 ventral midbrain (VM) dopaminergic neurons precursors. The best co-expression of LMX1A and FOXA2, crucial factors for inducing ventral midbrain fate, was obtained at day 12 of differentiation. After generating and characterizing the VM precursors, these cells were cultured in Neurobasal medium, 1% P/S and 2% B27-VitA with neurotrophic factors: 20 ng/ml of BDNF (450-02, Peprotech), 20 ng/ml of GDNF (450-10, Peprotech), 1 ng/ml of TGF-B3, (R&D Systems 243-B3), Ascorbic acid (Sigma A-4034), 0.5 mM of dbcAMP (D0627-25MG, SIGMA), and 5 µM of DAPT (565770; Calbiochem). On day 20, the precursors were split into wells previously coated with Poly Ornithine (15 µg/ml)/human Laminin (1 g/mL) and Fibronectin (2 µg/mL). Cells were differentiated for additional 15, 30, and 60 days, finally providing the timepoints states in the main text of 35, 50, and 80 days. Studied cultures were fixed with PFA 4% and characterized for VM specificity.

### Immunocytochemistry

The differentiated cultures were fixed with 4% PFA (15 min), washed three times with DPBS (15 min), then washed with either TBS1x (low triton protocol for vesicles specific antibodies) or with TBS1+ (for standard protein immunocytochemistry) 3 times for 15 min and then blocked for 2 h with TBS++ with or without low triton. Primary antibodies were incubated for 48 h at 4 °C. Samples were then washed with TBS 1x/TBS+ (15 min) three times. The blocking was next repeated for 1 h at room temperature followed by 2 h incubation with the secondary antibodies (all at 1:200 dilution). The antibodies used are listed in Supplementary Table 4 in Supplementary Information. The samples were then washed with TBS 1x (15 min) three times and then incubated with nuclear staining DAPI (Invitrogen, dilution 1:5000) for 10 min. After washing twice the DAPI with TBX1x, samples were mounted with PVA:DABCO, dried for 2 h at room temperature, and stored at 4 °C until imaged. Samples were imaged using an SP5 confocal microscope (Leica®) and analyzed with FIJI® is Just ImageJTM®.

### Gene expression analysis using Human Neuropathology Panel

50 ng of total RNA per sample was prepared for analysis with a NanoString Human Neuropathology Panel chip. The assay was performed on an nCounter SPRINT Analysis System (Sanford Consortium for Regenerative Medicine Stem Cell Genomics Core, La Jolla) according to the manufacturer’s instructions. The nSolver software by NanoString was used to normalize gene expression data. ROSALIND software (OnRamp Bioinformatics, https://rosalind.onramp.bio/) was then used to interpret targeted gene expression data and to create heatmaps. Data was then analyzed by ROSALIND® (https://rosalind.onramp.bio/), with a HyperScale architecture developed by OnRamp BioInformatics, Inc. (San Diego, CA) to interpret targeted gene expression data and to create heatmaps. Read Distribution percentages, violin plots, identity heatmaps, and sample MDS plots were generated as part of the QC step. The limma R library^37^ was used to calculate fold changes and *p*-values. Clustering of genes for the final heatmap of differentially expressed genes was done using the PAM (Partitioning Around Medoids) method using the fpc R library (https://cran.r-project.org/web/packages/fpc/index.html) that takes into consideration the direction and type of all signals on a pathway, the position, role, and type of every gene, etc. Hypergeometric distribution was used to analyze the enrichment of pathways, gene ontology, domain structure, and other ontologies. Functional enrichment analysis of pathways, gene ontology, domain structure, and other ontologies was performed using HOMER^38^. Several database sources were referenced for enrichment analysis, including Interpro^39^, NCBI^40^, KEG^41,42^, MSigDB^43^, REACTOME^44^, and WikiPathways^45^. Enrichment was calculated relative to a set of background genes, relevant to the experiment. The transcriptomic data has been deposited in Gene Expression Omnibus (GEO) of the National Center for Biotechnology Information and are accessible through GEO Series accession number SE167335.

### Neuronal quantification during differentiation

Immunostaining images were analyzed using Fiji® software to quantify the percentage of TH/DAPI at day 35, 50, and 80, and TH/FOXA2 and TH/GIRK at day 50 and the presence of pyknotic nuclei at day 50 and 80. An average of 5 images was quantified for each ratio, and each differentiation was performed at least three times.

### Neurite quantification

Immunostaining images were analyzed with NeuronJ® software to quantify number of neurites per neuron and neurite length for TH+ cells. Each neuron was analyzed in NeuronJ® and each trace was automatically measured and organized in order to obtain information for every single cell. Immunostaining images were analyzed with NeuronJ® software to quantify number of neurites per neuron for MAP2+ cells. An average of five images and ten neurons per image were analyzed at each timepoint for TH+ and MAP2+ data for every iPSC-derived line.

### Calcium imaging assay

We used calcium fluorescence imaging^46–49^ to evaluate the differences in spontaneous activity between healthy and PD neurons. Calcium imaging allowed the monitoring of a large population of neurons, simultaneously and non-invasively (Fig. 2). Living neurons were incubated for 30 min in a solution that contained 3 ml of the recording medium (EM, consisting of 128 mM NaCl, 1 mM CaCl_2_, 1 mM MgCl_2_, 45 mM sucrose, 10 mM glucose, and 0.01 M Hepes; pH 7.4) and 4 µg/ml of the cell-permeant calcium-sensitive dye Fluo-8-AM. At the end of incubation, cultures were washed with 2 ml of fresh EM to remove residual Fluo-8 and transferred to a glass-bottom dish (Mattek) filled with EM for imaging. The dish was mounted on a Zeiss inverted microscope equipped with a CMOS camera (Hamamatsu Orca Flash 2.8) and an arc lamp for fluorescence. Greyscale images of spontaneous neuronal activity were acquired at 20 Hz for 15 min in a field of view of 2.8 × 2.1 mm^2^ that contained between 300 and 700 neurons. A bright-field image of the monitored region was taken at the end of the recording session for easier cell identification. Data was then analyzed with the custom software NETCAL, run on MatLab®, to extract the trains of neuronal activations, as follows. Regions of Interest (ROIs) corresponding to cell bodies that exhibited prototypical neuronal morphology were manually drawn on the bright-field images, and their fluorescence intensity as a function of time extracted (Fig. 2a). These fluorescence traces were then inspected to remove non-neuronal signals (either undifferentiated cells or glia). A Schmitt-trigger method^49^ was next used on the fluorescence traces to identify the timing of neuronal activations, finally procuring the set of spike trains for each neuron. The resulting data of neuronal network activity was visualized in the form of raster plots. Collective episodes of coherent activity (network bursts) appeared as the synchronous activation of a large fraction of the neurons in the network in a short time window.

### Determination of DA release levels

The supernatant collected from DA neurons differentiated from CTR (SP11), PD1 (SP12), PD2 (SP13), and isoPD1 (SP12 wt/wt) for 50 and 80 days (D50 and D80), was harvested and kept directly at −80 °C until the moment of analysis. Before their analysis, medium samples were previously deproteinized with 50 µl of homogenization medium (100 mL miliQ H2O, 100 mg of sodium metabisulphite (S-1516, Sigma), 10 mg EDTA-Na (E5134, Sigma), 100 mg cysteine (C-4022, Sigma), and 3.5 mL de HClO4 concentrated (Scharlau, 70%); centrifuged at 15,000 rpm during 30′ at 4 °C and the supernatant was filtered (Millex-HV 0.45 μm, Millipore) for a posterior HPLC injection. The concentration of dopamine (DA) in supernatant (SN) samples was determined by an HPLC system consistent of a Waters 717plus autosampler (Waters Cromatografia), a Waters 515 pump, a 5 μm particle size C18 column (100 × 46 mm, Kinetex EVO, Phenomenex), and a Waters 2465 amperometric detector set at an oxidation potential of 0.75 V. The mobile phase consisted of 0.15 M NaH_2_PO_4_.H_2_O, 0.57 mM 1-octane sulfonic acid, 0.5 mM EDTA (pH 2.8, adjusted with phosphoric acid), and 7.4% methanol and was pumped at 0.9 ml/min. The total sample analysis time was 50 min and the DA retention time was 3.94 min. The detection limit was 2–3 fmol (injection volume 60 µl). Corresponding dopamine metabolite content was normalized to protein concentration determined previously by Bradford method detection.

### Average neuronal activity and global network activity (GNA)

The average neuronal activity quantified the degree of spontaneous activity in the recordings and was determined by counting the number of activations per neuron and minute, averaging afterward across neurons and realizations of the same line and timepoint. The GNA quantified the capacity of the network to exhibit collective synchronous events (*bursts*), and was determined by, first, counting the neurons that activated together in a sliding window of 1 s in length (corresponding to 20 image frames) without repetition and, second, by normalizing the count with the number of active neurons in the network. GNA thus varied between 0 (no activity) and 1 (full network activation). Bursts appeared in the GNA data as sharp peaks. The higher the GNA amplitude, the higher the number of participating neurons in the burst (Fig. 2c).

### Ratio of extreme events of network bursting

They corresponded to those GNA episodes in which neuronal participation was much higher than average and relatively to control (CTR) cultures. To compute the number of extreme events, GNA data were analyzed as follows. First, for each recording, background activity was determined by iteratively removing all peaks in the GNA signal that exceed by two standard deviations the average GNA value, procuring a background activity that was typically around GNA ≅ 0.05 (5% of the network). The non-background peaks of the recording were then ascribed as truly bursting episodes, with amplitudes that were typically above GNA = 0.1. Second, all the peak amplitudes observed in CTR cultures were pooled together and the average peak amplitude *A*_CTR_ and standard deviation *SD*_CTR_ determined. Then, for each realization and condition (CTR, isoPD1, PD1, and PD2), those bursting peaks that were above *A*_CTR_ + 2*SD*_CTR_ were considered as ‘extreme events’. The ‘ratio of extreme events’ *R*_EE_ was then determined, for each realization, as the ratio between the number of extreme peaks and the total number of observed peaks. Since this definition sets the CTR cultures as reference, some of the CTR realizations exhibited *R*_EE_ close to 0, while PD1 or PD2 realizations exhibited *R*_EE_ close to 1.

### Effective connectivity

Since the number of neurons varied among realizations, all connectivity analyses were carried out in networks with randomly chosen 340 neurons, the minimum population in all experiments. Also, to ensure that connectivity and network analyses reflected the impact of bursting behavior, only those recording with at least 10 bursting events were used for connectivity inference. Causal relationships among pairs of neuronal spike trains were inferred using a modified version of the Generalized Transfer Entropy (GTE)^50^. Briefly, given a pair of spike trains corresponding to neurons X and Y, an effective connection was established between X and Y whenever the information contained in X significantly increased the capacity to predict future states of Y (Granger causality). For the actual estimation of the effective connectivity, binarized time series (‘1’ for the presence of a spike, ‘0’ for absence) were constructed and computed in the fast implementation of GTE run in MatLab. Instant feedback was present, and Markov Order was set as 2. The actual GTE estimate was then compared with those from the joint distribution of all inputs to Y and all outputs to X, setting a connection as significant whenever the GTE estimate exceeded the mean + 1 standard deviations of the joint distribution. This threshold was considered optimal to capture the effective interactions among neurons during bursting episodes, which is the key dynamic characteristic separating CTR and isoPD cultures from PD ones. All network measures and connectivity statistics were computed with this threshold condition. However, for visualization purposes only, the GTE data shown in the functional matrices and network maps were thresholded at mean + 2.5 standard deviations, which procured the top 10% strongest links. In either case, the GTE scores were finally set to 0 (absence of connection) or 1 (connection present), shaping directed yet unweighted connectivity matrices. In all network maps, the directionality of the connections was not shown for clarity. Also, for clarity of language, the term ‘functional’ was used instead of ‘effective’ throughout the description of results.

### Functional communities

GTE connectivity data was analyzed, for each culture realization, to obtain the number of functional communities and their interrelation. A functional community corresponds to groups of neurons that are more connected within themselves than with the rest of network. They were detected using a fast implementation of the Louvain’s algorithm on the most significant connected component (Brain Connectivity Toolbox)^51^. Communities were visualized as boxes along the diagonal of the functional connectivity matrices. The strength of a community, i.e., how isolated it is from the rest of the network, was asserted through the community statistic Q. The larger Q, the higher the tendency of the network to split into characteristic communities. Q = 0 corresponds to the situation in which the network is highly integrated and the only community is the network itself, while Q = 1 corresponds to the extreme case in which all neurons are disconnected from one another and there are as many communities as neurons.

### Cumulative distribution of connections and Kullback–Leibler divergence

The effective connectivity matrix was analyzed to extract the distribution of connections p(k), i.e., the normalized histogram of the number of neurons having k connections. This distribution was transformed into the ‘cumulative distribution function’ CDF(k), which provides the probability that a neuron has many connections less or equal than k. The divergence between two CDF distributions *P* and *S* was quantified through the Kullback–Leibler divergence $D_{{\mathrm{KL}}}\left( {P||S} \right) = \mathop {\sum }\nolimits_i P\left( i \right)\ln \frac{{P\left( i \right)}}{{S\left( i \right)}}$, using the function KLDiv.m in MatLab. Significant statistical differences between P and Q were analyzed using the Kolmogorov–Smirnov test.

### In silico model

The model of Compte et al.^52^ was used to simulate a network of excitatory, inhibitory, and dopaminergic neurons. The model incorporated soma and synaptic dynamics as well as noise in the form of Poissonian trains of excitatory pre-synaptic potentials. Network construction was set by placing on a bidimensional space the same neurons as the ones observed experimentally in the form of ROIs, but limited to a randomly chosen population of 300 neurons, and axons grew using biologically-realistic rules as described^53,54^. Simulations were run for the equivalent of 10 min in the experiments, and 4 realizations were carried out for each pruning condition (no pruning, 10%, 30, and 50% pruned neurites). Raster plots of network activity were analyzed identically as in the experiments to compute the ratio of extreme events, the effective connective, and CDFs. Full details of the model are provided in Supplementary Information.

### Reporting summary

Further information on research design is available in the Nature Research Reporting Summary linked to this article.

## Supplementary information

Supplementary Information

Reporting Summary

Supplementary Data 1

## Data Availability

The authors declare that the main data supporting the findings of this study are available within the article and its Supplementary Information files. Extra data are available from the corresponding author upon request.
