# Supplementary material for: Parkinson’s disease patient-specific neuronal networks carrying the LRRK2 G2019S mutation unveil early functional alterations that predate neurodegeneration
Source: NPJ Parkinsons Dis. 2021 Jul 2;7:55. doi: 10.1038/s41531-021-00198-3 (PMC8253775; doi:10.1038/s41531-021-00198-3)
Supplement: Supplementary file 1 — Supplementary Information [file 41531_2021_198_MOESM1_ESM.pdf]

SUPPLEMENTAL INFORMATION

Parkinson's disease patient-specific neuronal networks carrying the LRRK2 G2019S mutation unveil early functional alterations that predate neurodegeneration

Carola G.[1,2] *, Malagarriga D.[1,2] *, Calatayud C.[1,2,3]*, Pons-Espinal M.[1,2]*, Blasco-Agell L.[1,2], Richaud-Patin Y.[3], Fernandez- Carasa I.[1,2], Baruffi V.[1,2], Beltramone S.[1,2,4], Molina E.[5], Dell'Era P.[4], Toledo-Aral J.J.[6], Tolosa E.[7], Muotri A.R.[8], Garcia Ojalvo J.[9], Soriano J.[10,11] §, Raya A.[3,12,13] §, Consiglio A.[1,2,4,§]

1. Department of Pathology and Experimental Therapeutics, Bellvitge University Hospital-IDIBELL, 08908 Hospitalet de Llobregat, Spain.
2. Institute of Biomedicine (IBUB) of the University of Barcelona (UB), 08028 Barcelona, Spain.
3. Regenerative Medicine Program, Bellvitge Biomedical Research Institute (IDIBELL), and Program for Clinical Translation of Regenerative Medicine in Catalonia (P-CMRC), Hospital Duran i Reynals, Hospitalet de Llobregat, 08908 Barcelona, Spain.
4. Cellular Fate Reprogramming Unit, Department of Molecular and Translational Medicine, University of Brescia, Piazza del Mercato, 15, 25121 Brescia BS, Italy.
5. Sanford Consortium for Regenerative Medicine, UC San Diego, La Jolla, California, United States of America.
6. Instituto de Biomedicina de Sevilla (IBiS), Hospital Universitario Virgen del Rocío/CSIC/Universidad de Sevilla, Sevilla, Spain.
7. Department of Neurology, Hospital Clínic de Barcelona, Institut d'Investigacions Biomèdiques August Pi i Sunyer (IDIBAPS), University of Barcelona (UB), 08036 Barcelona, Spain.
8. Departments of Pediatrics and Cellular & Molecular Medicine, University of California San Diego, La Jolla, California, United States of America.
9. Department of Experimental and Health Sciences, Universitat Pompeu Fabra, Parc de Recerca Biomèdica de Barcelona, Barcelona, Spain.
10. Department de Física de la Matèria Condensada, Universitat de Barcelona, 08028 Barcelona, Spain.
11. Universitat de Barcelona Institute of Complex Systems (UBICS), 08028 Barcelona, Spain.
12. Centre for Networked Biomedical Research on Bioengineering, Biomaterials and Nanomedicine (CIBER-BBN), 28029 Madrid, Spain.
13. Institució Catalana de Recerca i Estudis Avançats (ICREA), 08010 Barcelona, Spain

* These authors contributed equally to this work.

§ Correspondence and requests for materials should be addressed to: J.S. (email: jordi.soriano@ub.edu) or A.R. (email: araya@idibell.cat) or A.C.(email: consiglio@ub.edu)

Supplementary Methods: *In silico* model

Neuronal dynamics

The computational model is based on that described by Sancristobal *et al.* (2016), with the modifications described below. The dynamical equations for the neuronal membrane voltage and for the ionic channels are based on those introduced by Compte *et al.* (2003). In this model, the somatic and dendritic voltages, V_s and V_d respectively, of either excitatory or dopaminergic neurons are described by the equations:

$$C_m A_s \frac{dV_s}{dt} = -A_s(I_L + I_{Na} + I_K + I_A + I_{KS} + I_{KNa}) - I_{syn,s} - g_{sd}(V_s - V_d),$$
$$C_m A_d \frac{dV_d}{dt} = -A_d(I_{Ca} + I_{KCa} + I_{NaP} + I_{AR}) - I_{syn,d} - g_{sd}(V_d - V_s),$$

where $I_{syn,s}$ and $I_{syn,d}$ are the synaptic impinging from upstream neurons on soma and dendrites, respectively. Somatic and dendritic compartments are linked by an electrical coupling of conductance $\mathrm{g_{sd}} = 1.75 \pm 0.1\mu\mathrm{S}$ such that both voltages are quickly synchronized. The synaptic conductances and $\mathrm{g_{sd}}$ are scaled by the surfaces of the soma, $A_s = 0.015\mathrm{mm}^2$, and the dendrites, $\mathrm{A_d} = 0.035\mathrm{mm}^2$. The membrane capacitance is $\mathrm{C_m} = 1\mu\mathrm{F/cm}^2$.

The currents through the potassium and sodium channels are, respectively,

$$I_K = g_K n^4(V - V_k),$$

$$I_{N\mathrm{a}} = g_{Na} m^3 \mathrm{h}(V - V_{Na}),$$

which depend on the time-varying probability x that a channel is open as

$$\frac{dx}{dt} = \phi[\alpha_x(V)(1-x) - \beta_x(V)x] = \frac{1}{\tau_x(V)}\phi|x_\infty - x|.$$

Here x represents dimensionless variables that are associated with potassium channel activation ($x \equiv n$), sodium channel activation (m), and sodium channel inactivation (h), respectively. ϕ is a temperature factor set to 1. Parameter values were taken from Compte *et al.*[2], as follows: $g_K = 10.5mS/cm^g_{Na} = 50mS/cm$ and $g_L = 0.0667 \pm 0.0067ms/cm^2$. The rate functions α_x and β_x are, for each n,m,h variable, given by

$$\alpha_n(V) = 0.01\frac{V+34}{1-e^{-(V+34)/10}},$$

$$\beta_n(V) = 0.125e^{-(V+44)/25},$$

$$\alpha_m(V) = 0.1\frac{V+33}{1-e^{-(V+33)/10}},$$
$$\beta_m(V) = 4e^{-(V+53.7)/12},$$

$$\alpha_h(V) = 0.07e^{-(V+50)/10},$$
$$\beta_h(V) = \frac{1.0}{1+e^{-(V+20)/10}}.$$

Due to the rapid activation of m, this variable can be replaced by its steady-state value $m_\infty = \alpha_m/(\alpha_m + \beta_m)$.

The leak current I_L is a passive channel described as $I_L = g_L(V - V_L)$.

The current I_A through the fast A-type potassium channel is described as $I_A = g_A m_\infty^3(V - V_K)$, with $g_A = 1mS/cm^2$. This current has its fast activation variable replaced by its steady-state

$$m_\infty(V) = \frac{1}{1+e^{-(V+50)/20}},$$

and with an inactivation variable governed by

$$h_\infty(V) = \frac{1}{1+e^{(V+80)/6}},$$

and $\tau_h = 15\ ms$.

The current I_{KS} for the non-inactivating potassium channel is described as $I_{KS} = g_{KS}m(V - V_{KS})$, with $g_{KS} = 0.576\ mS/cm^2$ and

$$m_\infty(V) = \frac{1}{1+e^{-(V+34)/6.5}},$$
$$\tau_m(V) = \frac{8}{e^{-(V+55)/30}+e^{(V+55)/30}}.$$

The current I_{NaP} for the persistent sodium channel is given by $I_{NaP} = g_{NaP} m_\infty^3(V - V_{NaP})$, with $g_{NaP} = 0.0686\ mS/cm^2$ and

$$m_\infty(V) = \frac{1}{1+e^{-(V+55.7)/7.7}}.$$

The current I_{AR} for the inward rectifying Potassium channel is described as $I_{AR} = g_{AR}h_\infty(V - V_K)$, with $g_{AR} = 0.0257\ mS/cm^2$ and

$$h_\infty(V) = \frac{1}{1+e^{(V+75)/4}},$$

The current I_{Ca} for the high-threshold Ca^{2+} channel is given by $I_{Ca} = g_{Ca} m_\infty^2(V - V_{Ca})$, with $g_{Ca} = 0.43ms/cm^2$ and

$$m_\infty(V) = \frac{1}{1+e^{-(V+20)/9}}.$$

Two more channels are considered, which depend on ionic concentrations: the Ca^{2+}-dependent potassium channel and the Na^+-dependent potassium channel:

$$I_{KCa} = g_{KCa}\frac{[Ca^{2+}]}{[Ca^{2+}]+K_D}(V - V_K),$$
$$I_{KNa} = g_{KNa}\omega_\infty([Na^+])(V - V_K),$$

respectively, where $K_D = 30\,mM$, $g_{KCa} = 0.57\,mS/cm^2$, $\omega_\infty([Na^+]) = 0.37/\left((1 + (38.7/[Na^+])^{3.5})\right)$ and $g_{KNa} = 1.33\,mS/cm^2$. Besides, the intracellular concentration of calcium follows the dynamical equation:

$$\frac{d[Ca^{2+}]}{dt} = -\alpha_{Ca} A_d I_{Ca} - \frac{[Ca^{2+}]}{\tau_{Ca}},$$

where $\alpha_{Ca} = 5\,mM/(mA \cdot ms)$ and $\tau_{Ca} = 15\,ms$. The intracellular sodium concentration obeys the equation:

$$\frac{d[Na^+]}{dt} = -\alpha_{Na}(A_s I_{Na} + A_d I_{NaP}) - \left(R_{pump} + D\chi_{OU}(t)\right)\left(\frac{[Na^+]^3}{[Na^+]^3 + 15^3} - \frac{[Na^+]_{eq}^3}{[Na^+]_{eq}^3 + 15^3}\right),$$

where $\alpha_{Na} = 10 \pm 2 mM/(mA \cdot ms)$, $R_{pump} = 0.008 \pm 0.0018\,mM/ms$ and $[Na^+]_{eq} = 9.5\,mM$. We note that the sodium dynamics obeys the stoichiometry of the Na^K pump, which releases three sodium ions for each potassium ion brought inside the neuron. The reversal potentials of the different channels are $V_K = -100\,mV$, $V_{Na} = 55\,mV$, $V_{Ca} = 120\,mV$ and $V_L = -60.95 \pm 0.3\,mV$.

For inhibitory neurons, only the somatic compartment is modelled, with its membrane voltage following the dynamical equation

$$C_m A_i \frac{dV}{dt} = -A_i(I_L + I_{Na} + I_K) - I_{SYN,i},$$

where $A_i = 0.02\,mm^2$. The current term $I_{SYN,i}$ stands for the sum of both the excitatory and inhibitory currents coming from presynaptic neurons. Contrary to the excitatory neurons, the inhibitory ones are described with the minimum neuronal model for action potential generation. The sodium, potassium and leak currents follow the same formalism as in the above excitatory neuronal model with the following α and β rate functions:

$$\alpha_n(V) = 0.05\frac{V+34}{1-e^{-(V+34)/10}},$$
$$\beta_n(V) = 0.625e^{-(V+44)/80},$$

for the gating variable n;

$$\alpha_m(V) = 0.5\frac{V+35}{1-e^{-(V+35)/10}},$$
$$\beta_m(V) = 20e^{-(V+60)/18},$$

for the gating variable m; and

$$\alpha_h(V) = 0.35e^{-(V+58)/20},$$
$$\beta_h(V) = \frac{5.0}{1+e^{-(V+28)/10}},$$

for the gating variable h.

The temperature factor ϕ is also set to 1. The maximal conductances are $g_{Na} = 35 mS/cm^2$, $g_K = 9\,mS/cm^2$ and $g_L = 0.1025 \pm 0.0025\,mS/cm^2$, and reversal

potentials $V_K = -90\,mV$, $V_{Na} = 55\,mV$ and $V_L = -65 \pm 0.15\,mV$. All parameters are kept constant except in the cases given as mean $\pm$ SD. SD corresponds to the standard deviation of a Gaussian distributed parameter over the population. The Nernst equation establishes the relation between the reversal potential of a ionic channel V_{ion} (or equilibrium potential at which there is no net flow of ions across the channel) and the ratio C_o/C_i between the external and internal concentration of the corresponding ion, as

$$V_{ion} = \frac{RT}{zF} ln\left(\frac{C_o}{C_i}\right),$$

where $R = 8.314\,\mathrm{J/(K \cdot mol)}$ is the universal gas constant, T the temperature in Kelvin, z the number of elementary charges of the ion and F the Faraday constant (96.845 C/mol).

Synaptic dynamics

We considered two types of excitatory synapses, mediated by AMPA and NMDA (Wang 1999):

$$I_{AMPA} = g_{AMPA}s(t)\left(V - E_{syn}\right),$$
$$I_{NMDA} = g_{NMDA}s(t)\left(V - E_{syn}\right)\frac{1}{1+[Mg^{2+}]e^{-0.062V/3.57}},$$

where the reversal synaptic potential is taken as $E_{syn} = 0\,mV$ and the extracellular magnesium concentration is $[Mg^{2+}] = 1.0mM$. The g_{AMPA} and g_{NMDA} peak conductances are given in **Supplementary Table 1** below.

The time-dependent part of the corresponding conductances, $s(t)$, follows the first-order kinetics:

$$\frac{ds}{dt} = \phi(\alpha_s x(1 - s) - s/\tau_s),$$

with

$$\frac{dx}{dt} = \phi\left(\alpha_x \sum_j \delta\left(t - t_j\right) - x/\tau_x\right),$$

where $\phi = 1$ and the temporal time constants are shown in **Supplementary Table 2** below.

The inhibitory neurons are mediated by GABA, with corresponding synaptic current equal to $I_{GABA} = g_{GABA}s(t)\left(V - E_{syn}\right)$, where $E_{syn} = -70mV$. The g_{GABA} peak conductance is given in **Supplementary Table 1** below and $s(t)$ again follows first-order kinetics:

$$\frac{ds}{dt} = \alpha_i \sum_j \delta\left(t - t_j\right)(1 - s) - \frac{1}{\tau_i}s,$$

where $\alpha_i = 0.9\,\mathrm{ms}^{-1}$ and $\tau_i = 10\,ms$.

Supplementary Table 1: Synaptic conductances for all the possible connections.

Synapse	Conductance on inhibitory (nS)	Conductance on excitatory (nS)
GABA	240	480
Recurrent AMPA	2.25	7.35
Recurrent NMDA	3.0	8.0
External AMPA	2.25	7.35
External NMDA	2.0	8.0

Supplementary Table 2: Synaptic time constants for AMPA and NMDA synapses.

Synapse	α_x	τ_x	α_s	τ_s
AMPA	$1.0\ \mathrm{ms}^{-1}$	$0.05\ ms$	$1.0\ ms^{-1}$	$2.0\ ms$
NMDA	$1.0\ \mathrm{ms}^{-1}$	$2.0\ ms$	$1.0\ ms^{-1}$	$80\ ms$

Noise contribution

Additionally, all neurons received a heterogeneous Poisson train of excitatory pre-synaptic potentials with an event rate (mean = $50\ spikes/s$) that varies following an Ornstein- Uhlenbeck process $\chi_{OU}(t)$ of amplitude $D = 500\ spikes/s$. This train of potentials mimics the synaptic input coming from other neuronal regions connected to the modelled network. A noisy realization is obtained following the Heun algorithm (Mannella 1997):

$$\chi_{OU}(t+h) = \chi_{OU}(t)e^{-h/\tau} + \sqrt{D\frac{(1-e^{-2h/\tau})}{2\tau}}\,\mathrm{u}(t+h)$$

where $\chi_{OU}(0) = \left(1/\sqrt{2\pi}\right)\mathrm{u}(0)$, $u(t)$ a Gaussian white noise and τ a correlation time with value $\tau = 16\ ms$.

Connectivity

Neuronal networks were simulated on a bi-dimensional space, with their spatial location matching the position of the extracted ROIs in the analyzed cultures. The *in silico* neurons were physically simulated as circular cell bodies (somas, diameter $\Phi = 15mm$) and with axons that grew in random directions following a quasi-straight path. The final length of a given axon was given by the Rayleigh distribution, of the form:

$$p(\mathrm{l}) = \frac{1}{\sigma_\mathrm{l}^2} e^{\frac{-\mathrm{l}}{2\sigma}},$$

where $\sigma_\mathrm{l}^2 = 900\mu m$ is the variance of the distribution and its value was chosen so that the average axonal length matched the value $\langle \mathrm{l} \rangle \simeq 1.1\ mm$ measured in experiments. Experimental observations show that axons do not grow straight but with a fluctuating orientation. To mimic this condition realistically, the following algorithm to position each axon was applied. The total length l was split into small segments $\Delta \mathrm{l} = 10\mu m$ long. The first segment was placed at the end of the neuronal cell body, and with an orientation that followed a uniform angular distribution. The i-th segment was then placed at the end of

the previous one, and oriented following a Gaussian distribution around the previous segment given by

$$p(\theta_i) = \frac{1}{\sqrt{2\pi\sigma_\theta^2}} e^{-\frac{(\theta_i-\theta_{i-1})^2}{2\sigma_\theta^2}},$$

where θ_{i-1} is the angle between the segment $i-1$ and the $x-y$ plane. The variance σ_θ^2 was chosen to obtain a long persistence length (typically $\sigma_\theta \sim 15$ degrees). The growing process was then repeated until all segments were laid down.

The description of the network was completed by setting up the dendritic tree, which was implemented as a disk of diameter ϕ_d centred at the neuron's soma (Orlandi et al., 2013). The diameter was drawn from a Gaussian distribution with mean $\mu = 300\ \mu m$ and variance $\sigma^2 = 40\ \mu m$. Network connectivity after placing somas and axons was set as follows. First, a connection could be potentially established when the axon of a given neuron intersected the dendritic tree of any other neuron. Second, those neurons that fulfilled this geometric condition connected to one another with probability α. This connection probability was considered to be independent of the overlapping length between the axon and the dendritic tree that was intersected. Thus, for $\alpha = 1$, any axon that crossed a dendritic tree established a connection. In large cortical structures where the geometrical distribution is well known, α falls in the range $0.1-0.2$. However, in neuronal cultures, α is larger due to the reduced connectivity and dimensionality. In the present study, α took random values in the range $0.3-1$. The whole network connectivity that resulted from this geometric construction was stored in an adjacency matrix $\boldsymbol{A} = \{a_{ij}\}$, where a_{ij} identified a connection $i \rightarrow j$.

Numerical methods

The model has been integrated using the Heun algorithm (Manella 1997), with a time step of 0.05 ms.

References cited in the Supplementary Methods

Sancristobal et al. 2016. Collective stochastic coherence in recurrent neuronal networks. *Nature Physics* **12**:881.

Compte et al. 2003. Cellular and Network Mechanisms of Slow Oscillatory Activity (<1 Hz) and Wave Propagations in a Cortical Network Model. *J Neurophysiol* **89**:2707.

Wang X-J. 1999. Synaptic basis of cortical persistent activity: the importance of NMDA receptors to working memory. *J Neurosci* **19**:9587.

Mannella R, in Supercomputation in Nonlinear and Disordered Systems: Algorithms, Applications and Architectures, edited by L. Vázquez, F. Tirando, and I. Martín (World Scientific, Singapore, 1997).

Orlandi et al. 2013. Noise focusing and the emergence of coherent activity in neuronal cultures. *Nature Physics* **9**:58.

Supplementary Table 3. List of oligonucleotides used during gene edition

Gene	Purpose	Name	Target sequence
LRRK2	LRRK2(G2019S)-KI	L2_TALEN_L1	TGCCATCATTGCAAAGATT
		L2_TALEN_R1	TACAGCAGTACTGAGCAAT
	LRRK2(G2019S)-Correction	L2sel_gRNA_Fwd	CACCGTGTCTCATAATTCTATCTTC
		L2sel_gRNA_Rv	AAACGAAGATAGAATTATGAGACAA
	LRRK2 HDR donor template	LRRK2_5HA_KpnI_Fwd	AAGGTACCCCTTAATATCTAACATGATTAGG
		LRRK2_5HA_XhoI_Rv	AACTCGAGAAGATAGAATTATGAGACAGAC
		Rex1_LoxP_SalI_Fwd	AAGTCGACATAACTTCGTATAGCATACATTATACGAAGTTATGACCGATTCCTCCCGATAAG
		NeoR_LoxP_BamHI_Rv	AAGGATCCATAACTTCGTATAATGTATGCTATACGAAGTTATTAAGATACATTGATGAGTTTGGA
		LRRK2_3HA_SpeI_Fwd	AAACTAGTCAGGATGGATAACCACTGAC
		LRRK2_3HA_NotI_Rv	AAGCGGCCGCTCCCTAAAGATAGAGTGTTCC
TH	THrep-KI	TH_gRNA1_Fwd	CACCGGACGCCGTGCACCTAGCCAA
		TH_gRNA1_Rv	AAACTTGGCTAGGTGCACGGCGTCC
	TH HDR donor template	TH_5HA_KpnI_Fwd	AAGGTACCCCATCCGTGAGAAGGACCC
		TH_5HA_ApaI_Rv	AAGGGCCCGCCAATGGCACTCAGCGCA
		2A_ApaI_Fwd	AAGGGCCCGCCACGAACTTCTCTCTGTTAA
		mOrange_XhoI_RV	AACTCGAGCTACTTGTACAGCTCGTCC
		TH_3HA_SpeI_Fwd	AAACTAGTGTGCACGGCGTCCCTGAG
		TH_3HA_XbaI_Rv	AATCTAGATCCCCATCAGCACACATGC
Molecular screening		Check_LRRK2_5HA_Out_Fwd	TCGTGATTGCGTGGGTC
		Check_LRRK2_3HA_Out_Rv	GCAGGAAACGAACTAGAACC
		Check_TH_5HA_Out_F	GAAAACCGACCCCTGGTTG
		Check_TH_3HA_Out_R	GTGTTGGGTGCTCTCTCTG
		P2A_Screen_Rv	CTTCCACGTCTCCTGCTTG
		NeoR_Screen_Rv	CTTATCGGGAGGAATCGGTC
		bGH-pA_Screen_Fwd	CCCGTCTGTTGTGTGACTC

Supplementary Table 4. List of antibodies used in our studies

Antibody	Host	Reference	Dilution	Low Triton
MAP-2 (Polyclonal IgG)	Rabbit	Santa Cruz sc-20172	1:1000	NO
GIRK2 (Polyclonal IgG)	Rabbit	Sigma P8122	1:40	NO
FOXA2 (Polyclonal IgG)	Goat	R&D Systems (AF2400)	1:250	NO
LMX1A (Polyclonal IgG)	Rabbit	Millipore (AB10533)	1:1000	NO
Engrailed (D-20) (Polyclonal IgG)	Goat	Santa Cruz (sc-46101)	1:200	NO
Nestin (Polyclonal IgG)	Rabbit	Chemicon (AB5922)	1:250	NO
α-synuclein (Monoclonal IgG1)	Mouse	BD, 610787	1:500	SI
TH (Polyclonal IgG)	Sheep	Pel-Freez, P60101-0	1:500	NO
TH (Polyclonal IgG)	Rabbit	Santa Cruz, sc-14007	1:250	NO
DAT (Monoclonal IgG2aκ)	Rat	Chemicon (MAB369)	1:300	SI
RFP (Polyclonal IgG)	Rabbit	Abcam (ab34771)	1:400	NO
Alexa Fluor 488, Mouse IgG	Donkey	Jackson 715-545-150	1:200	N/A
Cy™3, Rabbit IgG	Donkey	Jackson 711-165-152	1:200	N/A
Alexa Fluor 647, Sheep IgG	Donkey	Jackson 713-605-147	1:200	N/A
Cy™3, Rat IgG	Donkey	Jackson 712-165-153	1:200	N/A
Cy™2, Rabbit IgG	Donkey	Jackson 711-225-152	1:200	N/A
Cy™3, Mouse IgG	Donkey	Jackson 715-165-151	1:200	N/A

Figure S1

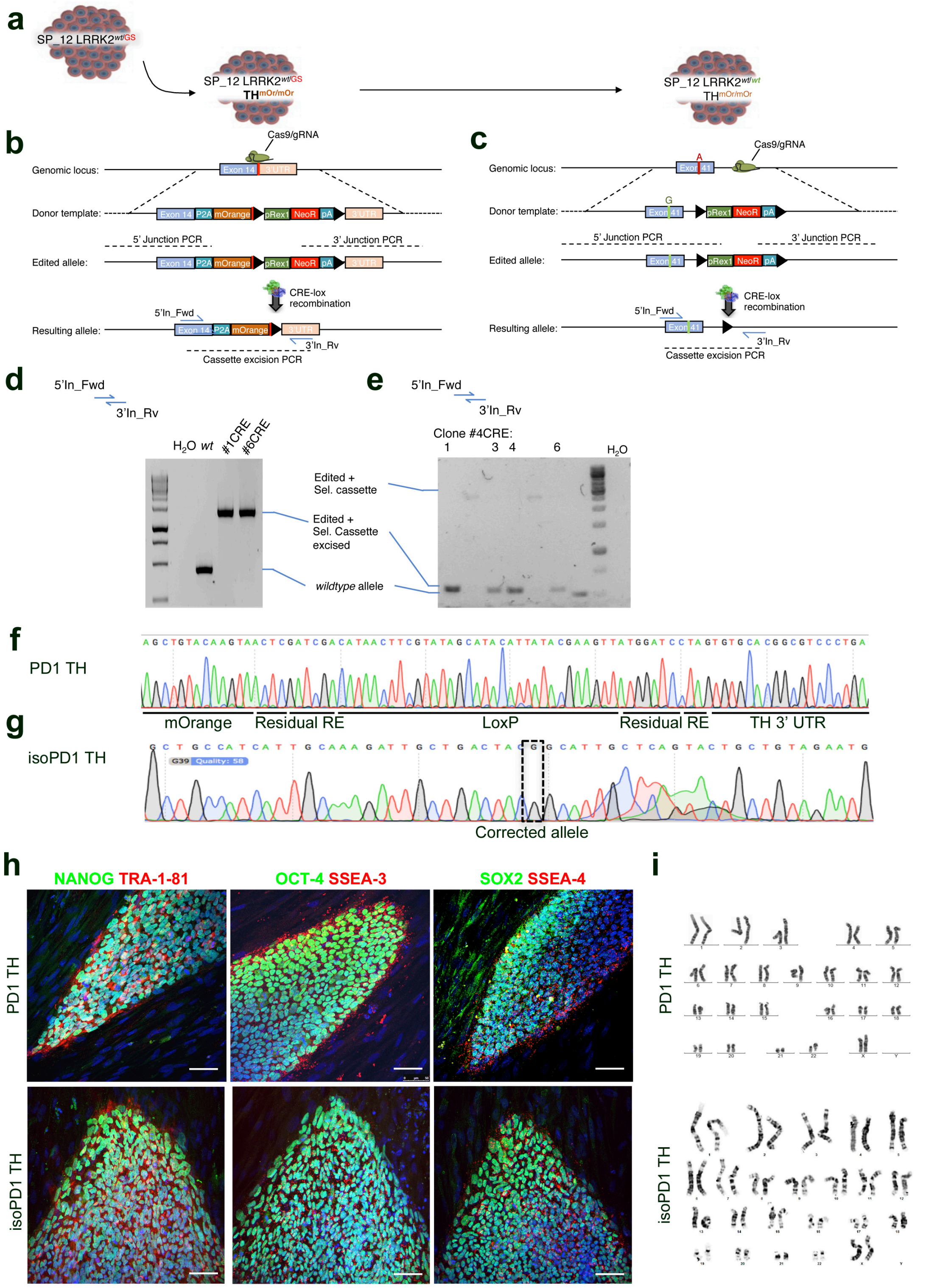

Supplementary Fig. 1: Generation of a knock-in fluorescent reporter in the *TH* gene in LRRK2 G2019S SP12 iPSC line and subsequent correction of the mutation using CRISPR/Cas9. a Sequence of the gene edition in the parental SP 12 LRRK2 G2019S hiPSC line. **b-c** Scheme describing the recombination steps during the edition process

of the TH (a) and the LRRK2 (c) gene. Blue arrows represent the primers used for the PCR screening procedure. Black triangles represent LoxP sites surrounding the selection cassette. **d** PCR analysis of the correctly targeted clones to confirm proper P2A-mOrange cassette integration and selection cassette excision in the control iPSC line. **e** Subclone screening of a correctly targeted LRRK2 G2019S-corrected clone to confirm selection cassette excision after CRE recombinase transfection. Note that after the excision of the selection cassette, the remaining LoxP site confers an increase in size of >30bp in the edited compared to the *wild type* allele. **f** Sanger sequencing confirmed successful excision of the LoxP site-flanked cassette in the selected SP 12 TH-mOrange reporter line. **g** Sanger sequencing confirmed the presence of the *wild type* allele in the p.2019 position in the selected SP12 TH-mOrange LRRK2$^{wt/wt}$ line. **h** Immunofluorescence analysis of representative colonies of the SP12 TH-mOrange and SP12 TH-mOrange LRRK2$^{wt/wt}$ lines staining positive for the pluripotency-associated markers NANOG, OCT4 and SOX2 (green) and TRA-1-81, SSEA3 and SSEA4 (red). Scale bar, 50 µm. **i** Normal karyotype of the SP12 TH-mOrange and SP12 TH-mOrange LRRK2$^{wt/wt}$ lines.

Figure S2

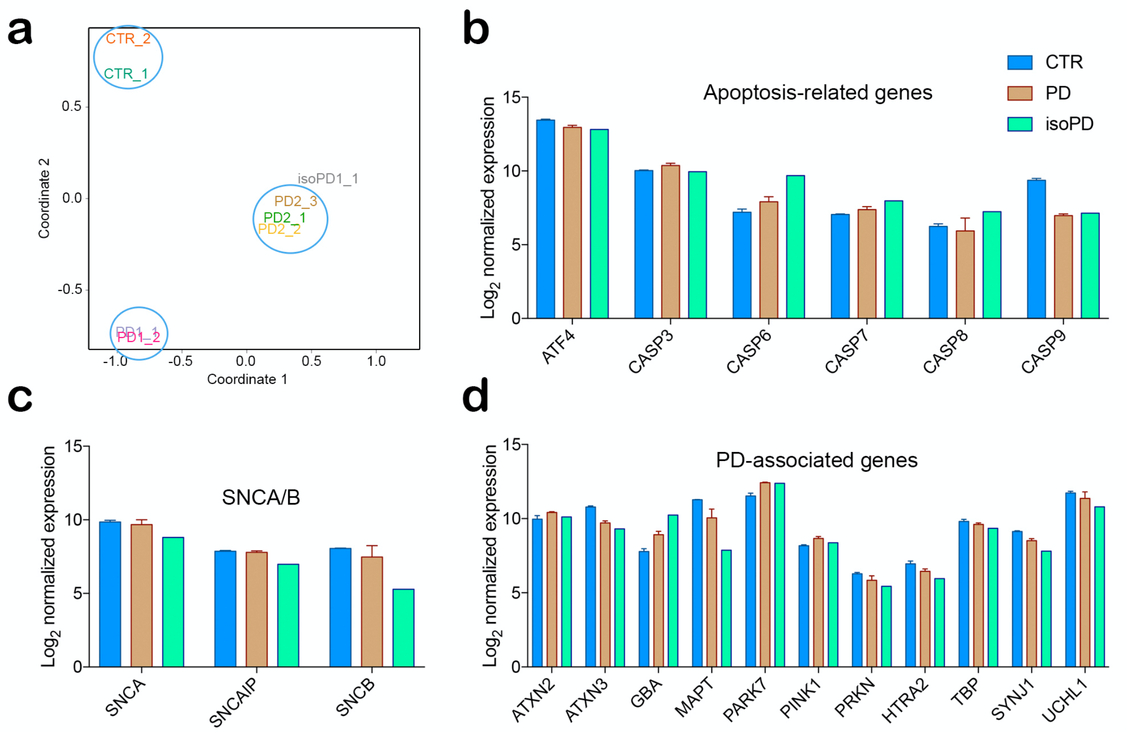

Supplementary Fig. 2: Gene expression profiling of iPSC-derived DAn. a Multidimensional scaling (MDS) plot showing a visual representation of the proximities among gene expression profiles of neuronal cultures at D50 derived from CTR (SP11), PD1 (SP12), PD2 (SP13), and gene-edited isoPD1 (SP12 wt/wt) iPSC. Shown are two (CTR and PD1) or three (PD2) independent experiments, except for the gene-edited isogenic PD1 line, for which only one RNA preparation passed the quality control and could be analyzed. Gene expression profiles clustered by iPSC cell line (indicated by circles), rather than by condition. **b-d** Log2 normalized expression of specific genes measured with the NanoString Human Neuropathology Panel and particularly relevant for PD-related neurodegeneration, including those related to apoptosis (b), synuclein (c), and previously associated with PD (d), in CTR, PD and isoPD cultures at D50, shown for a better visualization of the absence of changes in expression across conditions.

Figure S3

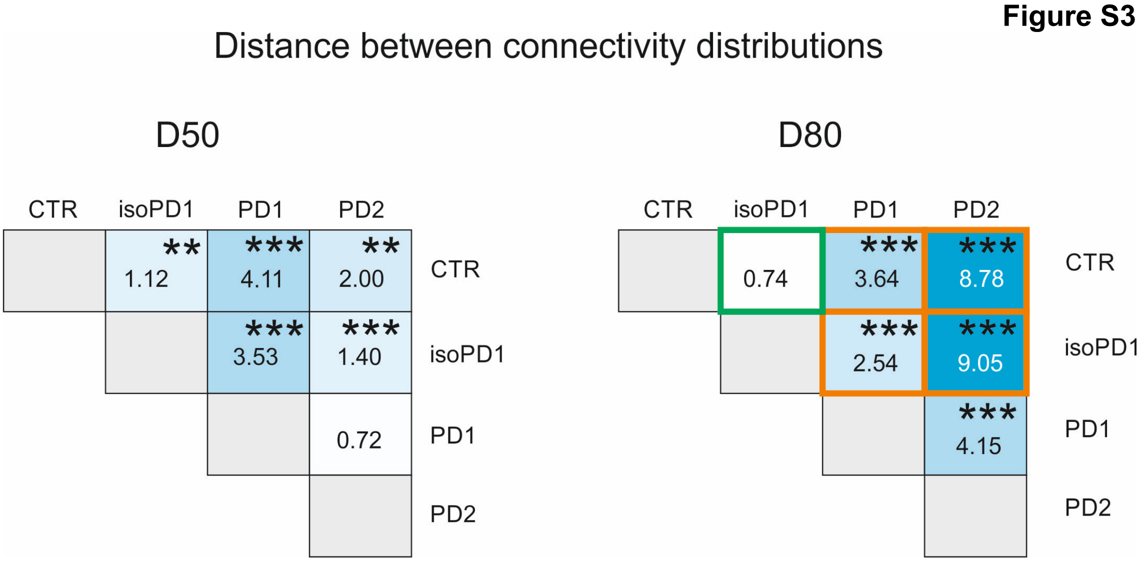

Supplementary Fig. 3: Statistical variability between the cumulative distribution functions (CDFs) of functional connectivity in experimental data. The analysis corresponds to the data shown in Figs 3d-e, and compares the CDFs of all cell lines at D50 (left) and D80 (right). The variability is quantified through the Kullback-Leibler divergence. The tables show the values of the divergence. The higher the value, the larger the separation between CDF curves. Colour strength is proportional to the divergence values for clarity. Significance is established according to a Kolmogorov-Smirnov test (*: $p<0.05$; **: $p<0.01$; ***: $p<0.001$). At D50, the two PD lines are very close to one another and are not significantly different among themselves. However, they strongly differ from CTR and isoPD1. There is a difference between CTR and isoPD1, but not as strong as the other cases. At D80, the two PD lines have slightly departed from one another but, more importantly, are substantially different from CTR and isoPD1 (orange highlights). CTR and isoPD1 are very similar to one another and exhibit no significant difference among themselves (green highlight).

Figure S4

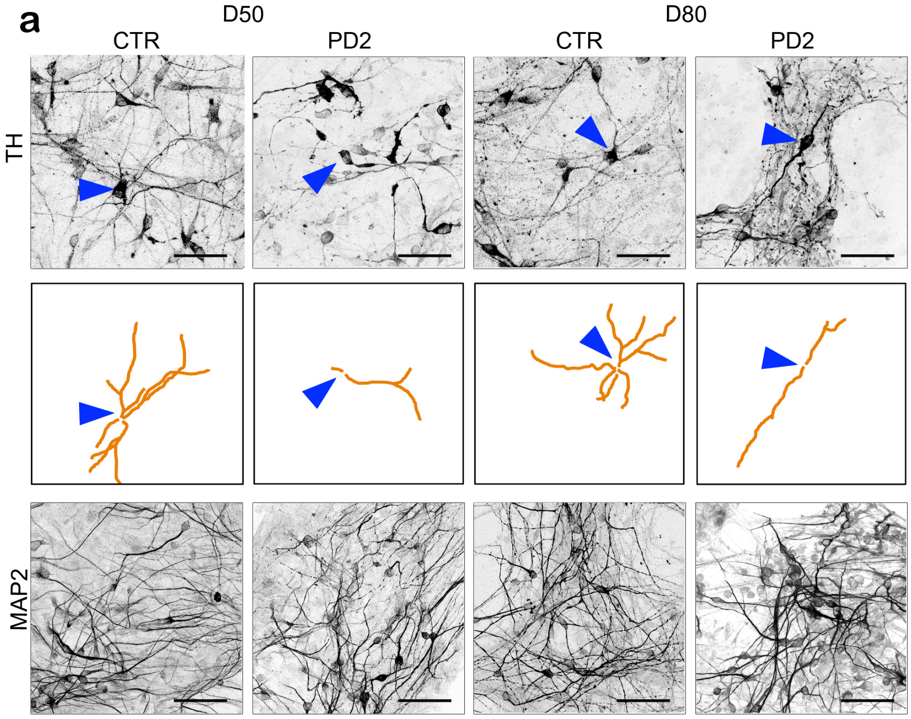

Supplementary Fig. 4: Biological confirmation of the *in silico* hypothesis. a CTR (SP11) and PD2 (SP13) TH+ neuronal processes are traced (orange) to determine the number and the structure of neurites at D50 (left panels) and D80 (right panels). The top panels for each cell line show the original image of TH+ neurons. A blue triangle highlights a representative traced neuron shown in the middle panels. The bottom panels for each cell line show MAP2+ neuronal processes. Scale bar is 50 µm in all images.

Figure S5

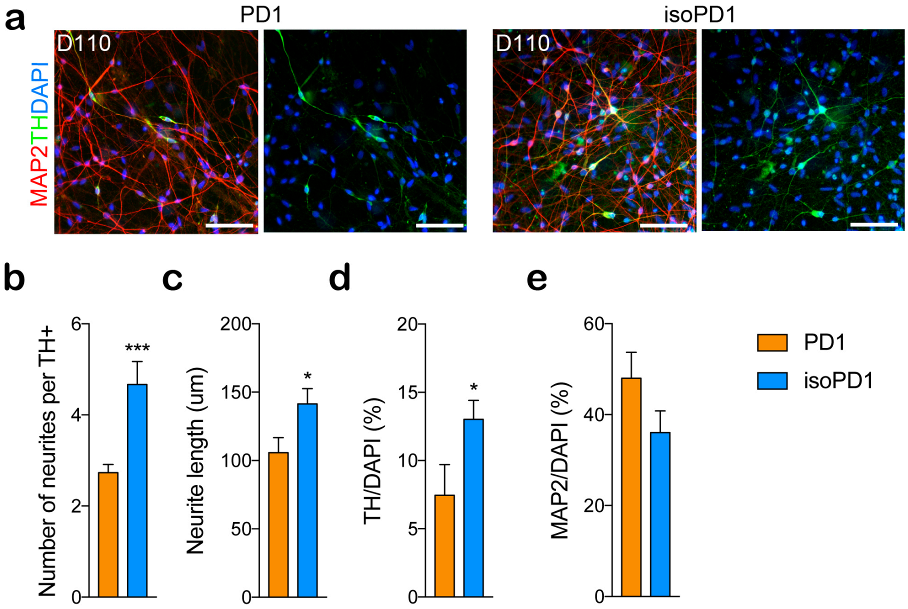

Supplementary Fig. 5: Characterization of the neurodegeneration phenotype on gene-edited isoPD1 (SP12 wt/wt) line. a Representative image of PD1 (SP12) and isoPD1 (SP12 wt/wt) neuronal culture at D110 showing the expression of TH and MAP2 markers. **b-c** The quantification of the number of TH neurites (b) and neurite length (c) show significant differences between isoPD1 and PD1. **d** Percentage of TH+ neurons over DAPI. **e** Percentage of MAP2+ neurons over DAPI. * p-value<0.05; *** p-value<0.001 (Multiple t-test analysis).
