# Supplementary material for: Parkinson’s disease patient-specific neuronal networks carrying the LRRK2 G2019S mutation unveil early functional alterations that predate neurodegeneration
Source: NPJ Parkinsons Dis. 2021 Jul 2;7:55. doi: 10.1038/s41531-021-00198-3 (PMC8253775; doi:10.1038/s41531-021-00198-3)
Supplement: Supplementary file 2 — Reporting Summary [file 41531_2021_198_MOESM2_ESM.pdf]

nature research

Corresponding author(s): NPJPARKD-00463R2

Last updated by author(s): 05/24/2021

Reporting Summary

Nature Research wishes to improve the reproducibility of the work that we publish. This form provides structure for consistency and transparency in reporting. For further information on Nature Research policies, see our Editorial Policies and the Editorial Policy Checklist.

Statistics

For all statistical analyses, confirm that the following items are present in the figure legend, table legend, main text, or Methods section.

n/a	Confirmed	
☐	☒	The exact sample size (*n*) for each experimental group/condition, given as a discrete number and unit of measurement
☐	☒	A statement on whether measurements were taken from distinct samples or whether the same sample was measured repeatedly
☐	☒	The statistical test(s) used AND whether they are one- or two-sided<br>*Only common tests should be described solely by name; describe more complex techniques in the Methods section.*
☐	☒	A description of all covariates tested
☐	☒	A description of any assumptions or corrections, such as tests of normality and adjustment for multiple comparisons
☐	☒	A full description of the statistical parameters including central tendency (e.g. means) or other basic estimates (e.g. regression coefficient) AND variation (e.g. standard deviation) or associated estimates of uncertainty (e.g. confidence intervals)
☐	☒	For null hypothesis testing, the test statistic (e.g. *F*, *t*, *r*) with confidence intervals, effect sizes, degrees of freedom and *P* value noted<br>*Give P values as exact values whenever suitable.*
☒	☐	For Bayesian analysis, information on the choice of priors and Markov chain Monte Carlo settings
☐	☒	For hierarchical and complex designs, identification of the appropriate level for tests and full reporting of outcomes
☐	☒	Estimates of effect sizes (e.g. Cohen's *d*, Pearson's *r*), indicating how they were calculated

Our web collection on statistics for biologists contains articles on many of the points above.

Software and code

Policy information about availability of computer code

Data collection	Image acquisition for Calcium analysis: Hokawo 2.10 (Hamamatsu Photonics).<br>Immunostaining images were obtained using a SP5 confocal microscope (Leica).
Data analysis	Calcium Imaging Assay: Data was then analyzed with the custom software NETCAL, run on MatLab®<br>Transcriptomic analysis: Data was analysed using ROSALIND ONRAMP Version 3.19.0.7.<br>Immunostaining images were analyzed using Fiji® software Version 2.0.0-rc-69/1.53i.<br>Statistical significance tests were conducted in Prism, Origin 9.0 and Matlab. Numerical simulations were run in Python and Matlab

For manuscripts utilizing custom algorithms or software that are central to the research but not yet described in published literature, software must be made available to editors and reviewers. We strongly encourage code deposition in a community repository (e.g. GitHub). See the Nature Research guidelines for submitting code & software for further information.

Data

Policy information about availability of data

All manuscripts must include a data availability statement. This statement should provide the following information, where applicable:

- Accession codes, unique identifiers, or web links for publicly available datasets
- A list of figures that have associated raw data
- A description of any restrictions on data availability

The authors declare that the main data supporting the findings of this study are available within the article and its Supplementary Information files. Extra data are available from the corresponding author upon request.
Code data analysis was carried out with the custom software NETCAL, run in Matlab, and freely available at https://github.com/orlandi/netcal.

Transcriptomic analysis is submitted to GEO database <https://www.ncbi.nlm.nih.gov/geo/query/acc.cgi?acc=GSE167335>
GEO accession number: GSE167335.

Field-specific reporting

Please select the one below that is the best fit for your research. If you are not sure, read the appropriate sections before making your selection.

☒ Life sciences ☐ Behavioural & social sciences ☐ Ecological, evolutionary & environmental sciences

For a reference copy of the document with all sections, see nature.com/documents/nr-reporting-summary-flat.pdf

Life sciences study design

All studies must disclose on these points even when the disclosure is negative.

Sample size	A minimum of 3 independent experiments were performed for each experiment. Differences among groups were evaluated through an ANOVA analysis with multiple comparisons, and comparisons between two groups through Student's t-test, using Prism (Mac OS X), Origin 9.0 and Matlab. Error bars represent mean±SD. The threshold for significance was established at a p-value $p<0.05$.
Data exclusions	Not applicable
Replication	Experimental replicates were performed to get enough statistical significance power in the analysis.
Randomization	The study did not include treatment groups. Control iPSC lines were compared to PD lines. Gene-edited corrected iPSC lines served as controls.
Blinding	Researcher were blinded during data analysis.

Reporting for specific materials, systems and methods

We require information from authors about some types of materials, experimental systems and methods used in many studies. Here, indicate whether each material, system or method listed is relevant to your study. If you are not sure if a list item applies to your research, read the appropriate section before selecting a response.

Materials & experimental systems

n/a	Involved in the study	
☐	☒	Antibodies
☒	☐	Eukaryotic cell lines
☒	☐	Palaeontology and archaeology
☒	☐	Animals and other organisms
☒	☐	Human research participants
☒	☐	Clinical data
☒	☐	Dual use research of concern

Methods

n/a	Involved in the study	
☒	☐	ChIP-seq
☒	☐	Flow cytometry
☒	☐	MRI-based neuroimaging

Antibodies

Antibodies used	MAP-2 (Polyclonal IgG) Rabbit. Santa Cruz Technologies. Cat. number sc-20172.<br>GIRK2 (Polyclonal IgG) Rabbit. Sigma-aldrich. Cat. number P8122.<br>FOXA2 (Polyclonal IgG) Goat. R&D Systems. Cat. number AF2400.<br>LMX1A (Polyclonal IgG) Rabbit. Millipore. Cat. number AB10533<br>Engrailed (D-20) (Polyclonal IgG) Goat. Santa Cruz Biotechnologies. Cat. number sc-46101.<br>Nestin (Polyclonal IgG) Rabbit. Chemicon. Cat. number AB5922.<br>α synuclein (Monoclonal IgG1) Mouse. BD Biosciences. Cat. number 610787.<br>TH (Polyclonal IgG) Sheep. Pel-Freez. Cat. number P60101-0.<br>TH (Polyclonal IgG) Rabbit. Santa Cruz Biotechnologies. Cat. number sc-14007.<br>DAT (Monoclonal IgG2aκ) Rat. Chemicon. Cat. number MAB369.<br>RFP (Polyclonal IgG) Rabbit. Abcam. Cat. number .<br>Alexa Fluor 488, Mouse IgG Donkey. Jackson ImmunoResearch. Cat. number 715-545-150.<br>Cy™3, Rabbit IgG Donkey. Jackson ImmunoResearch. Cat. number 711-165-152.<br>Alexa Fluor 647, Sheep IgG Donkey. Jackson ImmunoResearch. Cat. number 713-605-147.<br>Cy™3, Rat IgG Donkey. Jackson ImmunoResearch. Cat. number 712-165-153.<br>Cy™2, Rabbit IgG Donkey. Jackson ImmunoResearch. Cat. number 711-225-152.<br>Cy™3, Mouse IgG Donkey. Jackson ImmunoResearch. Cat. number 715-165-151.
Validation	MAP-2 (Polyclonal IgG) Rabbit. This antibody was validated as suitable for immunofluorescence in SK-N-SH cells as stated by the manufacturer.

GIRK2 (Polyclonal IgG) Rabbit. Has been tested and used in 5 studies according to manufacturer's datasheet.
FOXA2 (Polyclonal IgG) Goat. This antibody was validated as suitable for immunocytochemistry in Endoderm Differentiated BG01V Human Stem Cells as stated by the manufacturer.
LMX1A (Polyclonal IgG) Rabbit. Has been tested and used in 4 studies according to manufacturer's datasheet.
Engrailed (D-20) (Polyclonal IgG) Goat. Has been tested and used in 6 studies according to manufacturer's datasheet.
Nestin (Polyclonal IgG) Rabbit. Has been tested and used in 2 studies according to manufacturer's datasheet.
α synuclein (Monoclonal IgG1) Mouse. Has been tested and used in 5 studies according to manufacturer's datasheet.
TH (Polyclonal IgG) Sheep. This antibody was validated as suitable for immunostaining in rabbit retina as stated by the manufacturer.
TH (Polyclonal IgG) Rabbit. Has been tested and used in 23 studies according to manufacturer's website.
DAT (Monoclonal IgG2aκ) Rat. Has been tested and used in more than a 100 studies according to manufacturer's website.
RFP (Polyclonal IgG) Rabbit. Has been tested and used in 66 studies according to manufacturer's website.
